# Model-free detection of unique events in time series

**DOI:** 10.1038/s41598-021-03526-y

**Published:** 2022-01-07

**Authors:** Zsigmond Benkő, Tamás Bábel, Zoltán Somogyvári

**Affiliations:** 1 Department of Computational Sciences, Wigner Research Centre for Physics, Budapest, 1121 Hungary; 2 János Szentágothai Doctoral School of Neurosciences, Semmelweis University, Ullői road 26, Budapest, 1085 Hungary

**Keywords:** Computational biology and bioinformatics, Cardiology, Astronomy and planetary science, Mathematics and computing, Physics

## Abstract

Recognition of anomalous events is a challenging but critical task in many scientific and industrial fields, especially when the properties of anomalies are unknown. In this paper, we introduce a new anomaly concept called “unicorn” or unique event and present a new, model-free, unsupervised detection algorithm to detect unicorns. The key component of the new algorithm is the Temporal Outlier Factor (TOF) to measure the uniqueness of events in continuous data sets from dynamic systems. The concept of unique events differs significantly from traditional outliers in many aspects: while repetitive outliers are no longer unique events, a unique event is not necessarily an outlier; it does not necessarily fall out from the distribution of normal activity. The performance of our algorithm was examined in recognizing unique events on different types of simulated data sets with anomalies and it was compared with the Local Outlier Factor (LOF) and discord discovery algorithms. TOF had superior performance compared to LOF and discord detection algorithms even in recognizing traditional outliers and it also detected unique events that those did not. The benefits of the unicorn concept and the new detection method were illustrated by example data sets from very different scientific fields. Our algorithm successfully retrieved unique events in those cases where they were already known such as the gravitational waves of a binary black hole merger on LIGO detector data and the signs of respiratory failure on ECG data series. Furthermore, unique events were found on the LIBOR data set of the last 30 years.

## Introduction

Anomalies in time series are rare and non-typical patterns that deviate from normal observations and may indicate a transiently activated mechanism different from the generating process of normal data. Accordingly, recognition of anomalies is often important or critical, invoking interventions in various industrial and scientific applications.

Anomalies can be classified according to various aspects^1–3^. These non-standard observations can be point outliers, whose amplitude is out of range from the standard amplitude or contextual outliers, whose measured values do not fit into some context. A combination of values can also form an anomaly named a collective outlier. Thus, in the case of point outliers, a single point is enough to distinguish between normal and anomalous states, whilst in the case of collective anomalies, a pattern of multiple observations is required. Two characteristic examples of extreme events are black swans and dragon kings, distinguishable by their generation process^4,5^. Black swans are generated by a power law process and they are usually unpredictable by nature. In contrast, the dragon king, such as stock market crashes, occurs after a phase transition and it is generated by different mechanisms from normal samples making it more predictable. Both black swans and dragon kings are extreme events easily recognizable post-hoc (retrospectively), but not all the anomalies are so effortless to detect. Even post-hoc detection can be a troublesome procedure when the amplitude of the event does not fall out of the data distribution.

Although the definition of an anomaly is not straightforward, two of its key features include rarity and dissimilarity from normal data.

Most, if not all the outlier detection algorithms approach the anomalies from the dissimilarity point of view. They search for the most distant and deviant points without much emphasis on their rarity. In contrast, our approach is the opposite: we quantify the rarity of a state, largely independent of the dissimilarity.

Here we introduce a new type of anomaly, the unique event, which is not an outlier in the classical sense of the word: it does not necessarily lie out from the background distribution, neither point-wise nor collectively. A unique event is defined as a unique pattern that appears only once during the investigated history of the system. Based on their hidden nature and uniqueness one could call these unique events “unicorns” and add them to the strange zoo of anomalies. Note that unicorns can be both traditional outliers appearing only once or patterns that do not differ from the normal population in any of their parameters.

But how do you find something you’ve never seen before, and the only thing you know about is that it only appeared once?

The answer would be straightforward for discrete patterns, but for continuous variables, where none of the states are exactly the same, it is challenging to distinguish the really unique states from a dynamical point of view.

### Related works

Classical supervised, semi-supervised, and unsupervised strategies have been used to detect anomalies^1,6,7^ and recently deep neural networks^8–10^ were applied to detect extreme events^11–16^. Supervised outlier detection techniques can be applied to identify anomalies when labeled training data is available for both normal and outlier classes. Semi-supervised techniques also utilize labeled training data, but this is limited to the normal or the outlier class. Some of the semi-supervised methods do not need perfectly anomaly-free data to learn the normal class but allow some outlier-contamination even in the training data^17^. Model-based pattern matching techniques can be applied to detect specific anomalies with best results when the mechanism causing the anomaly is well known and simple^18^. However, when the background is less well known or the system is too complex to get analytical results (or to run detailed simulations), it is hard to detect even specific types of anomalies with model-based techniques due to the unknown nature of the waveforms. Model-free unsupervised outlier detection techniques can be applied to detect unexpected events from time series in cases when no tractable models or training data is available.

The closest concept to our unicorns in the anomaly detection literature is the discord, defined as the unique subsequence, which is the farthest from the rest of the (non-overlapping) time series^19^. Multiple model-free unsupervised anomaly detection methods have been built based on the discord concept^19,20^. Other unsupervised anomaly detection techniques, such as the Local Outlier Factor (LOF) algorithm^21^ are based on k Nearest Neighbor (kNN) distances. The LOF algorithm was also adapted to time series data by Oehmcke et al.^22^.

In the followings, we present a new model-free unsupervised anomaly detection algorithm to detect unicorns (unique events), that builds on nonlinear time series analysis techniques such as time delay embedding^23^ and upgrades time-recurrence based non-stationarity detection methods^24^ by defining a local measure of uniqueness for each point.

We validate the new method on simulated data, compare its performance with other model-free unsupervised algorithms^19–21^ and we apply the new method to real-world data series, where the unique event is already known.

## Methods

### Time delay embedding

To adapt collective outlier detection to time series data, nonlinear time series analysis provides the possibility to generate the multivariate state space from scalar observations. The dynamical state of the system can be reconstructed from scalar time series^25^ by taking the temporal context of each point according to Takens’ embedding theorem^23^. This can be done via time delay embedding:

1 $$\begin{aligned} X(t) = [ x(t), x(t+\tau ), x(t+2\tau ), \ldots x(t+(E-1)\tau )] \end{aligned}$$

where *X*(*t*) is the reconstructed state at time *t*, *x*(*t*) is the scalar time series. The procedure has two parameters: the embedding delay ($\tau$) and the embedding dimension (*E*).

Starting from an initial condition, the state of a dynamical system typically converges to a subset of its state space and forms a lower-dimensional manifold, called the attractor, which describes the dynamics of the system in the long run. If E is sufficiently big ($E > 2*d$) compared to the dimension of the attractor (*d*), then the embedded (reconstructed) space is topologically equivalent to the system’s state space, given some mild conditions on the observation function generating the *x*(*t*) time series are also met^23^.

As a consequence of Takens’ theorem, small neighborhoods around points in the reconstructed state-space also form neighborhoods in the original state space, therefore a small neighborhood around a point represents nearly similar states. This topological property has been leveraged to perform nonlinear prediction^26^, noise filtering^27,28^ and causality analysis^29–32^. Naturally, time delay embedding can be introduced as a preprocessing step before outlier detection (with already existing methods i.e. LOF) to create the contextual space for collective outlier detection from time series.

Besides the spatial information preserved in reconstructed state space, temporal relations in small neighborhoods can contain clues about the dynamics. For example, recurrence time statistics were applied to discover nonstationary time series^24,33^, to measure attractor dimensions^34–36^ and to detect changes in dynamics^37,38^.

### Temporal Outlier Factor

**Figure 1:**
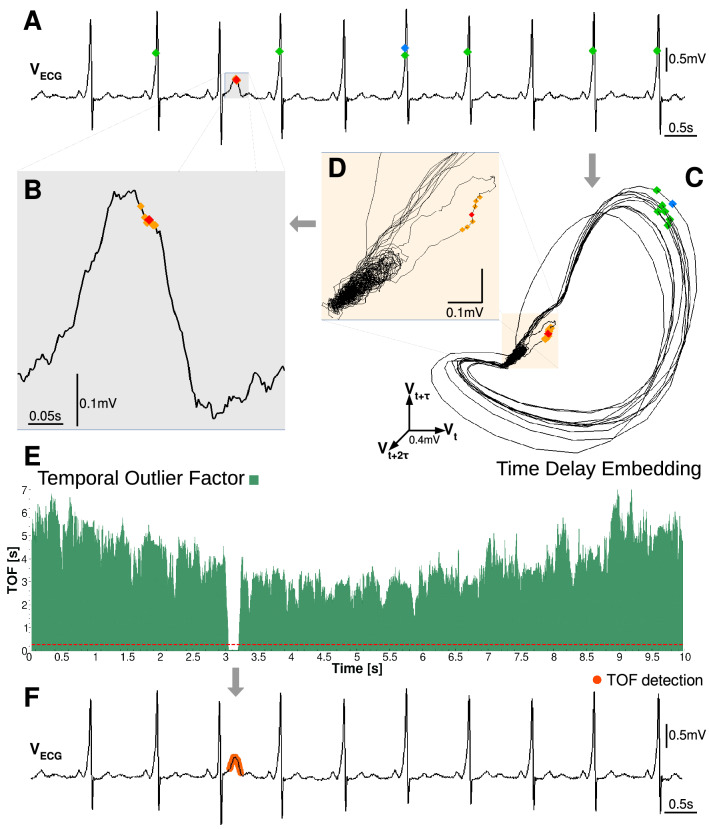
Schema of our unique event detection method and the Temporal Outlier Factor (TOF). (**A**) An ECG time series from a patient with Wolff-Parkinson-White Syndrome, a strange and unique T wave zoomed on graph (**B**). (**C**) The reconstructed attractor in the 3D state space by time delay embedding ($E=3, \tau =0.011\,{\text{s}}$). Two example states (red and blue diamonds) and their 6 nearest neighbors in the state space (orange and green diamonds respectively) are shown. The system returned several times back to the close vicinity of the blue state, thus the green diamonds are evenly distributed in time, on graph (**A**). In contrast, the orange state-space neighbors of the red point (zoomed on graph **D**) are close to the red point in time as well on graph (**A**). These low temporal distances show that the red point marks a unique event. (**E**) TOF measures the temporal dispersion of the *k* nearest state-space neighbors ($k=20$). The red dashed line is the threshold $\theta =0.28\,{\text{s}}$. Low values of TOF below the threshold mark the unique events, denoted by orange dots on the original ECG data on graph (**F**).

The key question in unicorn search is how to measure the uniqueness of a state, as this is the only attribute of a unique event. The simplest possible definition would be that a unique state appears only once in the time series. A problem with this definition arises in the case of continuous-valued observations, where almost every state is visited only once. Thus, a different strategy should be applied to find the unicorns. Our approach is based on measuring the temporal dispersion of the state-space neighbors. If state-space neighbors are separated by large time intervals, then the system returns to the same state time-to-time. In contrast, if all the state space neighbors are temporal neighbors as well, then the system never returned to that state again. This concept is shown on an example ECG data series from a patient with Wolff–Parkinson–White (WPW) Syndrome (Fig. 1). The WPW syndrome is due to an aberrant atrio-ventricular connection in the heart. Its diagnostic signs are shortened PR-interval and appearance of the delta wave, a slurred upstroke of the QRS complex. However, for our representational purpose, we have chosen a data segment, which contained one strange T wave with uniquely high amplitude (Fig. 1A).

To quantify the uniqueness on a given time series, the Temporal Outlier Factor (TOF) is calculated in the following steps (Fig. 1 and Fig. S1): firstly, we reconstruct the system’s state by time delay embedding (Eq. 1), resulting in a manifold, topologically equivalent to the attractor of the system (Fig. 1C-D and Fig. S1B).

Secondly, we search for the kNN in the state space at each time instance on the attractor. A standard choice for the distance metric is the Euclidean distance (Eq. 2).

2 $$\begin{aligned} d\left( X(t), X(t') \right) =\sqrt{\sum _{l=1}^{E}{(X_l(t)- X_l(t'))^2}} \end{aligned}$$

where *d* is the distance between the *X*(*t*) and $X(t')$ points, with $X_l$ as coordinate components in the reconstructed state space. We save the time index of the *k* nearest points around each sample to use it later on. Two examples are shown on Fig. 1C: a red and a blue diamond and their 6 nearest neighbors marked by orange and green diamonds respectively.

Thirdly, the Temporal Outlier Factor (*TOF*) is computed from the time indices of the kNN points (Fig. S1C):

3 $$\begin{aligned} \hbox {TOF}(t)=\root q \of {\frac{\sum _{i=1}^{k}{|t-t_{i}|}^{q}}{k}} \end{aligned}$$

where *t* is the time index of the sample point (*X*(*t*)) and $t_i$ is the time index of the *i*-th nearest neighbor in reconstructed state-space. Where $q\in {\mathcal {R}}^{+}$, in our case we use $q=2$ (Fig. 1E).

As a final step for identifying unicorns, a proper threshold $\theta$ should be defined for TOF (Fig. 1E, dashed red line), to mark unique events (orange dots, Fig. 1F).

TOF measures an expected temporal distance of the kNN neighbors in reconstructed state-space (Eq. 3), thus it has time dimension. A high or medium value of TOF implies that neighboring points in state-space were not close in time, therefore the investigated part of state-space was visited on several different occasions by the system. In our example, green diamonds on (Fig. 1C) mark states which were the closest points to the blue diamond in the state space, but were evenly distributed in time, on Fig. 1A. Thus the state marked by the blue diamond was not a unique state, the system returned there several times.

However a small value of TOF implies that neighboring points in state-space were also close in time, therefore this part of the space was visited only once by the system. On Fig. 1C,D orange diamonds mark the closest states to the red diamond and they are also close to the red diamond in time, on the (Fig. 1B). This results in a low value of TOF in the state marked by the red diamond and means that it was a unique state never visited again. Thus, small TOF values feature the uniqueness of sample points in state-space and can be interpreted as an outlier factor. Correspondingly, TOF values exhibit a clear breakdown at the time interval of the anomalous T wave (Fig. 1F).

The number of neighbors (*k*) used during the estimation procedure sets the minimal possible TOF value:

4 $$\begin{aligned} \mathrm{TOF}_{\mathrm{min}} =\sqrt{\frac{ \sum _{i=-k/2 }^{k/2} + k \bmod 2 }{{i^2}}k} \Delta t \end{aligned}$$

where $\lfloor k/2 \rfloor$ is the integer part of *k*/2, $\bmod$ is the modulo operator and $\Delta t$ is the sampling period.

The approximate maximal possible TOF value is determined by the length (*T*) and neighborhood size (*k*) of the embedded time series:

5 $$\begin{aligned} \mathrm{TOF}_{\mathrm{max}} = \sqrt{\frac{\sum _{i=0}^{k-1} (T - i \Delta t)^ 2 }{k} } \end{aligned}$$

TOF shows a time-dependent mean baseline and variance (Fig. 1E, Fig. S2) which can be computed if stationary activity without presence of anomaly is assumed. In this case, the time indices of the nearest points are evenly distributed along the whole time series. The approximate mean baseline is a square-root-quadratic expression, it has the lowest value in the middle and highest value at the edges (see exact derivation for continuous time limit and $q=1$ in the Supporting Information, Figs. S2-S3):

6 $$\begin{aligned}{}&\sqrt{\left\langle \hbox {TOF}_{\mathrm{noise}}\left( t\right) ^{2} \right\rangle } =\sqrt{t^2 - t T + \frac{T^2}{3}} \end{aligned}$$

7 $$\begin{aligned}&\mathrm{VAR} \left( {\mathrm{TOF}^{2}_{\mathrm{noise}}} \left( t \right) \right) =\frac{1}{k} \left( \frac{t^5 + (T-t)^5}{5 T} - \left( t^2 - tT + \frac{T^2}{3} \right) ^2 \right) \end{aligned}$$

Based on the above considerations, imposing a threshold $\theta$ on $TOF_{k}$ has a straightforward meaning: it sets a maximum detectable event length (*M*) or vice versa:

8 $$\begin{aligned} \theta = \sqrt{ \frac{\sum _{i=0}^{k-1}{\left( M-i \Delta t\right) ^2}}{k}} \quad \bigg | \quad k \Delta t {\mathop {\le }\limits ^{!}} M \end{aligned}$$

where in the continuous limit, the threshold and the event length becomes equivalent:

9 $$\begin{aligned} \lim _{\Delta t \rightarrow 0}{\theta (M)} = M \end{aligned}$$

Also, the parameter *k* sets a necessary detection criteria on the minimal length of the detectable events: only events with length $M\ge k \Delta t$ may be detected. This property comes from the requirement that there must be at least k neighbors within the unique dynamic regime of the anomaly.

The current implementation of the TOF algorithm contains a time delay embedding, a *k*NN search, the computation of TOF scores from the neighborhoods, and a threshold application for it. The time-limiting step is the neighbor-search, which uses the scipy cKDTree implementation of the kDTree algorithm^39^. The most demanding task is to build the data-structure; its complexity is $O(k n \log {n})$^40^, while the nearest neighbor search has $O(\log n)$ complexity.

Box 1: TOF analysis workflow

0. Preprocessing and applicability check
1. Time delay embedding (Eq. 1)
2. kNN Neighbor search (Eq. 2)
3. TOF score computation (Eq. 3)
4. Threshold application on TOF score to detect unicorns (Eq. 8).

### Previous methods to compare

We compare our method to widely used model-free, unsupervised outlier detection methods: the Local Outlier Factor (LOF) and two versions of discord detection algorithms^19,20^ (see SI). The main purpose of the comparison is not to show that our method is superior to the others in outlier detection, but to present the fundamental differences between the previous outlier concepts and the unicorns.

The first steps of all three algorithms are parallel: While TOF and LOF use time-delay embedding as a preprocessing step to define a state-space, discord detection algorithms reach the same by defining subsequences due to a sliding window. As a next step, state-space distances are calculated in all of the three methods, but with a slightly different focus. Both LOF and TOF search for the kNNs in the state-space for each time instance. As a key difference, the LOF calculates the distance of the actual points in state-space from their nearest neighbors and normalizes it with the mean distance of those nearest neighbors from their nearest neighbors, resulting in a relative local density measure. LOF values around 1 are considered the signs of normal behavior, while higher LOF values mark the outliers. While LOF concentrates on the densities of the nearest neighbors in the state-space, the discord concept is based on the distances directly. For each time instance, it searches for the closest, but temporary non-overlapping subsequence (state). This distance defines the distance of the actual state from the whole sequence and is called the matrix profile^41^. Finally, the top discord is defined as the state, which is the most distant from the whole data sequence by this means. Besides this top discord, any predefined number of discords can be defined by finding the next most distant subsequence which does not overlap with the already found discords.

The only parameter of this brute force discord detection algorithm is the expected length of the anomaly, which is given as the length of the subsequences used for the distance calculation. Senin et al.^20,42^ extended Keogh’s method by calculating the matrix profile for different subsequence lengths, then normalizing the distances by the length of the subsequences, and finally choosing the most distant subsequence according to the normalized distances. Through this method, Senin’s algorithm provides an estimation of the anomaly length as well. Both Keogh’s and Senin’s algorithm can be implemented in a slower but exact way by calculating all the distances, can be called as brute force algorithm or fastening them by using the Symbolic Aggregate approXimation (SAX) method. In our comparisons, Keogh’s brute force method was calculated exactly while SAX was used for Senin’s algorithm only.

### Simulated data series for validation

We tested the TOF method on various types of simulated data series to demonstrate its wide applicability. These simulations are examples of deterministic discrete-time systems, continuous dynamics, and a stochastic process.

**Figure 2:**
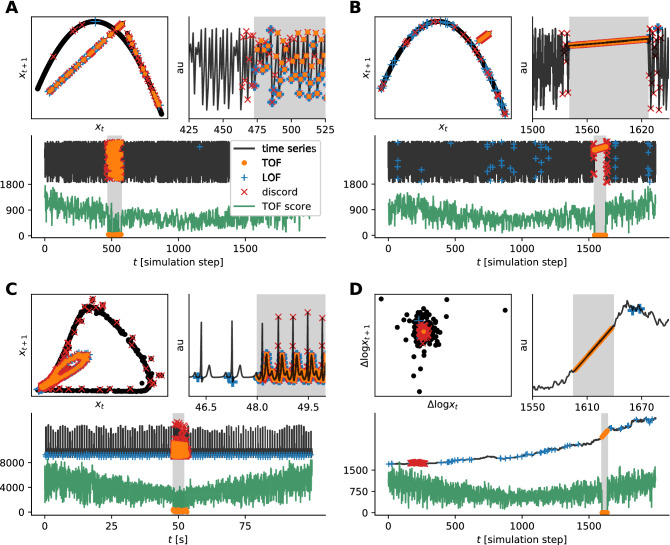
Detection examples on simulated time series with anomalies of different kinds. (**A**) Logistic map time series with tent-map anomaly. (**B**) Logistic map time series with linear anomaly. (**C**) Simulated ECG time series with tachycardia. (**D**) Random walk time series with linear anomaly, where TOF was measured on the discrete-time log derivative ($\Delta log x_t$). Each subplot shows an example time series of the simulations (black) in arbitrary units and in three forms: Top left the return map, which is the results of the 2D time delay embedding and defines the dynamics of the system or its 2D projection. Bottom: Full length of the simulated time series (black) and the corresponding TOF values (green). Shaded areas show anomalous sections. Top right: Zoom to the onset of the anomaly. In all graphs, the outliers detected by TOF, LOF, and Keogh’s brute force discord detection algorithms are marked by orange dots, blue plus, and red x signs respectively. While anomalies form clear outliers on A and B, D shows an example where the unique event is clearly not an outlier, but it is located in the center of the distribution. All the three algorithms detected the example anomaly well in case A, TOF, and discord detected well the anomalies in B and C cases, but only TOF was able to detect all the four anomaly examples.

We simulated two datasets with deterministic chaotic discrete-time dynamics generated by a logistic map^43^ ($N=2000$, 100–100 instances each) and inserted variable-length ($l=20$–200 step) outlier-segments into the time series at random times (Fig. 2A,B). Two types of outliers were used in these simulations, the first type was generated from a tent-map dynamics (Fig. 2A) and the second type was simply a linear segment with low gradient (Fig. 2B) for simulation details see the Supporting Information (SI). The tent map demonstrates the case, where the underlying dynamics is changed for a short interval, but it generates a very similar periodic or chaotic oscillatory activity (depending on the parameters) to the original dynamics. This type of anomaly is hard to distinguish by the naked eye. In contrast, a linear outlier is easy to identify for a human observer but not for many traditional outlier detection algorithms. The linear segment is a collective outlier and all of its points represent a state that was visited only once during the whole data sequence, therefore they are unique events as well.

As a continuous deterministic dynamics with realistic features, we simulated electrocardiograms with short tachycardic periods where beating frequency was higher (Fig. 2C). The simulations were carried out according to the model of Rhyzhii and Ryzhii^44^, where the three heart pacemakers and muscle responses were modeled as a system of nonlinear differential equations (see SI). We generated 100 s of ECG and randomly inserted 2–20 s long faster heart-rate segments, corresponding to tachycardia ($n=100$ realizations).

Takens’ time delay embedding theorem is valid for time series generated by deterministic dynamical systems, but not for stochastic ones. In spite of this, we investigated the applicability of time delay embedded temporal and spatial outlier detection on stochastic signals with deterministic dynamics as outliers. We established a dataset of multiplicative random walks ($n=100$ instances, $T=2000$ steps each) with randomly inserted variable length linear outlier segments ($l=20$–200, see SI). As a preprocessing step, to make the random walk data series stationary, we took the log-difference of time series as is usually the case with economic data series (Fig. 2D).

### Model evaluation metrics

TOF and LOF calculate scores on which thresholds should be applied to reach final detections. In contrast, the discord detection algorithms do not apply a threshold on the matrix profile values but choose the highest peak as a top discord. The effectiveness of TOF and LOF scores to distinguish anomalous points from the background can be evaluated by measuring the Area Under Receiver Operator Characteristic Curve^45^ (ROC AUC). The ROC curve consists of point pairs of True Positive Rate (TPR, recall) and False Positive Rate (FPR) parametrized by a threshold ($\alpha$, Eq. 10).

10 $$\begin{aligned} ROC(\alpha ) := \left( \mathrm{FPR}(\alpha ), \mathrm{TPR}(\alpha ) \right) \end{aligned}$$

where $\alpha \in [-\infty , \infty ]$. The area under the ROC curve can be computed as the Riemann integral of the TPR in the function of FPR on the (0, 1) interval.

This evaluation method considers all the possible thresholds, thus providing a threshold-independent measure of the detection potential for a score, where 1 means that a threshold can separate all the anomalous points from the background. Thus, we applied ROC AUC to evaluate TOF and LOF scores on the four datasets mentioned above with fixed embedding parameters $E=3$ and $\tau =1$ and determined its dependency on the neighborhood size ($k=1$–200) that was used for the calculations.

After choosing the optimal neighborhood parameter which maximises the ROC AUC values, precision, recall, and $\mathrm{F}_1$ score were used to evaluate the detection performance of the methods on the simulated datasets:

The precision metrics measures the ratio of true positive hits among all the detections:

11 $$\begin{aligned} \mathrm{precision}(\alpha ) = \frac{\mathrm{true} \, \mathrm{positives}(\alpha )}{\mathrm{true} \, \mathrm{positives}(\alpha ) + \mathrm{false} \, \mathrm{positives}(\alpha )} \end{aligned}$$

The recall evaluates what fraction of the points to be detected were actually detected:

12 $$\begin{aligned} \mathrm{recall}(\alpha ) = \frac{\mathrm{true} \, \mathrm{positives}(\alpha )}{\mathrm{true} \, \mathrm{positives}(\alpha ) + \mathrm{false} \, \mathrm{negatives}(\alpha )} \end{aligned}$$

$\mathrm{F}_1$ score is the harmonic mean of precision and recall and it provides a single scalar to rate model performance:

13 $$\begin{aligned} F_1(\alpha ) = 2 \, \frac{\mathrm{precision}(\alpha ) \times \mathrm{recall}(\alpha )}{\mathrm{precision}(\alpha ) + \mathrm{recall}(\alpha )} \end{aligned}$$

where the optimal the threshold ($\alpha$) were chosen to correspond to the actual mean number of anomalous points, or the expected length of the anomaly.

We implemented these steps in the python programming language (python3), the software is available at github.com/phrenico/uniqed. A detailed description of the data generation process and analysis steps can be found in the Supporting Information.

## Results

### Validation and comparison on simulated data series

**Figure 3:**
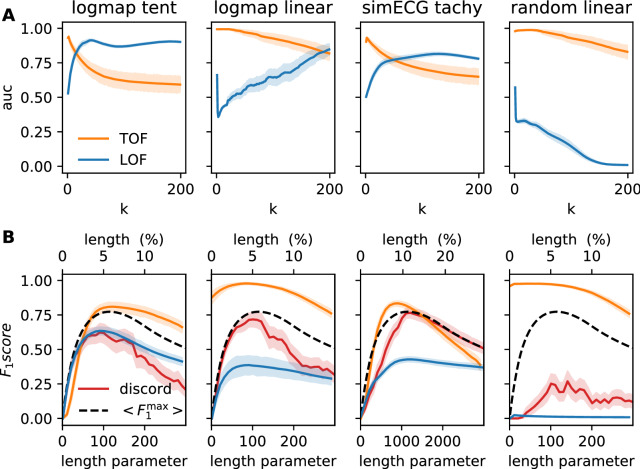
Performance evaluation of TOF, LOF, and Keogh’s discord detection algorithms on four simulated datasets. (**A**) Mean Receiver Observer Characteristic Area Under Curve (ROC AUC) score and SD for TOF (orange) and LOF (blue) are shown as a function of neighborhood size (*k*). TOF showed the best results for small neighborhoods. In contrast, LOF showed better results for larger neighborhoods in the case of the logistic map and ECG datasets but did not reach reasonable performance on random walk with linear outliers. (**B**) Mean $\mathrm{F}_1$ score for TOF (orange), LOF (blue), and Keogh’s discord detection (red) algorithms as a function of the expected anomaly length (for TOF) given in either data percentage (for LOF) or window length parameter (for discord). Black dashed lines show the theoretical maximum of the mean $\mathrm{F}_1$ score for algorithms with prefixed detection numbers or lengths (LOF and discord), but this upper limit does apply for TOF. The $\mathrm{F}_1$ score of TOF was very high for the linear anomalies and slightly lower for logistic map—tent map anomaly and ECG datasets, but it was higher than the $\mathrm{F}_1$ score of the two other methods and their theoretical limits in all cases. Note, that the only comparable performance was shown by discord detection on ECG anomaly, while neither algorithms based on discord nor LOF were able to detect the linear anomaly on random background.

Figure 3A shows the performance of the two methods in terms of mean ROC AUC and SD for $n=100$ realizations. TOF produced higher maximal ROC AUC than LOF in all four experimental setups. The ROC AUC values reached their maxima at small *k* neighborhood sizes in all of the four cases and decreased with increasing *k* afterward. In contrast, LOF resulted in reasonable ROC AUC values in only three cases (logmap-tent anomaly, logmap-linear anomaly, and ECG tachycardia), and it was not able to distinguish the linear anomaly from the random walk background at all. The ROC AUC values reached their maxima at typically higher *k* neighborhood size in the instances where LOF worked (Table 1).

In order to evaluate the final detection performance, as well as the type of errors made and the parameter dependency of these algorithms, $\mathrm{F}_1$ score, precision and recall were computed for all four algorithms. $\mathrm{F}_1$ score is especially useful to evaluate detection performance in cases of highly unbalanced datasets as in our case, see Methods.

As TOF showed the best performance in terms of ROC AUC with lower *k* neighborhood sizes, the $\mathrm{F}_1$ scores were calculated at a fixed $k=4$ neighborhood forming a simplex in the 3-dimensional embedding space^29^. In contrast, as LOF showed stronger dependency on neighborhood size, the optimal neighborhood sizes were used for $\mathrm{F}_1$ score calculations. The brute force discord detection algorithm uses no separate neighborhood parameter, as it calculates all-to-all distances between points in the state space.

Three among the four investigated algorithms require an estimation of the expected length of the anomaly, however, this estimation becomes effective through different parameters within the different algorithms. In the case of LOF, the expected length of the anomaly can be translated into a threshold, which determines the number of time instances above the threshold. In the absence of this information, the threshold is hard to determine in any principled way. In the case of Keogh’s brute force discord detection algorithm, the length of the anomaly is the only parameter and no further threshold is required. Both LOF and Keogh’s algorithm find the predefined number of time instances exactly. While the discord finds them in one continuous time interval, LOF detects independent points along the whole data. The expected maximal anomaly length is necessary to determine the threshold in the case of TOF as well (Eq. 8). As Senin’s discord detection algorithm does not require predefined anomaly length, it was omitted from this test, and we calculated the $\hbox {F}_1$ score at the self-determined window length.

Figure 3B shows the mean $\mathrm{F}_1$ scores for $\hbox {n}=100$ realizations, as a function of the expected anomaly length, for the three algorithms and for all the four test datasets. Additionally, Fig. S8 shows the precision and the recall, which are the two constituents of the $\mathrm{F}_1$ score as a function of the expected anomaly length as well. The actual length of the anomalies was randomly chosen between 20 and 200 time steps for each realization in three of our four test cases and between 200 and 2000 time steps in ECG realizations, thus the effect of the expected length parameters was examined up to these lengths as well.

**Table 1:** Detection performance on simulations in terms of ROC AUC scores and the optimal neighborhood parameter *k*. Maximal mean ROC AUC values and the corresponding SDs are shown. LOF was able to distinguish tent map and linear outliers from logistic background and tachycardia from the normal rhythm with reasonable reliability but TOF outperformed LOF for all data series. Linear outliers can not be detected on random walk background by the LOF method at all, while TOF detected them almost perfectly. TOF reached its maximal performance mostly for low *k* values, while LOF required larger *k* for optimal performance on those three data series, on which it worked reasonably. While the ROC AUC was maximal at $k=30$ in the case of random walk with linear outlier, the performance was not significantly lower for lower *k* values.

Dataset	TOF		LOF	
	*k*	AUC	*k*	AUC
Logmap-tent	2	$\mathbf {0.939} \pm \mathbf {0.050}$	42	$0.913 \pm 0.042$
Logmap-linear	6	$\mathbf {0.994} \pm \mathbf {0.007}$	199	$0.847 \pm 0.213$
Sim ECG-tachy	2	$\mathbf {0.931} \pm \mathbf {0.039}$	129	$0.815 \pm 0.056$
Randwalk-linear	30	$\mathbf {0.988} \pm \mathbf {0.014}$	1	$0.572 \pm 0.015$

While it is realistic, that we only have a rough estimate on the expected length of the anomaly, it turns out, that the randomness in the anomaly length sets an upper bound (Fig. 3B, black dashed lines, Fig. S6), for the mean $\mathrm{F}_1$ scores for those algorithms, that work with an exact predefined number of detections i.e. the LOF and the Keogh’s discord detection. Although the expected length parameter and the randomness in the actual anomaly length affect the detection performance of TOF as well, they do not set a strict upper bound, as the number of detections is not in a one-to-one correspondence with the expected anomaly length.

For all the four test datasets, TOF algorithm reached higher maximal $\mathrm{F}_1$ scores than the LOF and Keogh’s discord detection method (Fig. 3B, Fig. S8, orange lines). The maximal $\mathrm{F}_1$ score was even higher than the theoretical limit imposed by the variable anomaly lengths to the other methods. Similar to the results on ROC AUC values, the performance of the TOF algorithm was excellent on the linear type anomalies and very good for the logmap-tent map and the simulated ECG-tachycardia datasets.

In contrast, the LOF algorithm showed good performance on the logmap-tent map data series and mediocre results on logmap-linear anomalies and on the ECG-tachycardia data series. The linear outlier on random walk background was completely undetectable for the LOF method (Fig. 3B, Fig. S8, blue lines).

Keogh’s discord detection algorithm displayed good $\mathrm{F}_1$ scores on three datasets, but weak results were given in case of the linear anomaly on the random walk background (Fig. 3B, Fig. S8, red lines).

The simulated ECG dataset was the only one, where any of the competitor methods showed comparable performance to TOF: Keogh’s brute force discord detection reached its theoretical maximum, thus TOF resulted in an only slightly higher maximal $\mathrm{F}_1$ score in an optimal range of the length parameter. If the expectation significantly overestimated the actual length, the results of discord detection were slightly better.

**Table 2:** Performance evaluation by $\mathrm{F}_1$, precision and recall scores on simulations. The optimal expected anomaly length parameter (M) in time steps, mean scores, and their standard deviations are shown for all methods and datasets; the highest scores are highlighted in bold. In case of TOF, $k=4$ neighbour number is used, while for LOF, the *k* resulted the best ROC AUC were used from Table 1: $k=42$ for logmap-tent map, $k=199$ for logmap-linear, $k=129$ for ECG tachycardia and $k=1$ for random walk-linear datasets. TOF resulted in the highest $\mathrm{F}_1$ scores and highest precision for all datasets and the highest recall in three of the four cases but the simulated ECG tachycardia, where Keogh’s brute force discord detection algorithm reached a slightly higher recall score. The only comparable performance was reached by Keogh’s discord detection algorithm on ECG tachycardia in terms of $\mathrm{F}_1$ score while LOF produced reasonable results on logmap-tent map anomaly series. Although Senin’s discord detection algorithm resulted in reasonable mean estimations for the lengths of the anomalies, its detection performance was worse than the other three algorithms.

Method	TOF	LOF	Keogh	Senin
Dataset	Logistic map—tent map			
Length (M)	121	91	91	$137.06 \pm 93.68$
$\mathrm{F}_1$	$\mathbf {0.810} \pm \mathbf {0.175}$	$0.635 \pm 0.141$	$0.624 \pm 0.329$	$0.002 \pm 0.016$
Precision	$\mathbf {0.920} \pm \mathbf {0.139}$	$0.702 \pm 0.231$	$0.720 \pm 0.387$	$0.002 \pm 0.014$
Recall	$\mathbf {0.734} \pm \mathbf {0.185}$	$0.659 \pm 0.149$	$0.586 \pm 0.337$	$0.003 \pm 0.019$
Dataset	Logistic map—linear			
Length (M)	81	91	101	$146.56 \pm 91.17$
$\mathrm{F}_1$	$\mathbf {0.978} \pm \mathbf {0.038}$	$0.387 \pm 0.353$	$0.717 \pm 0.273$	$0.267 \pm 0.358$
Precision	$\mathbf {0.978} \pm \mathbf {0.053}$	$0.382 \pm 0.366$	$0.766 \pm 0.332$	$0.220 \pm 0.308$
Recall	$\mathbf {0.981} \pm \mathbf {0.038}$	$0.459 \pm 0.428$	$0.752 \pm 0.289$	$0.370 \pm 0.473$
Dataset	Sim ECG—tachycardia			
Length (M)	910	1110	1210	$1128.04 \pm 1024.98$
$\mathrm{F}_1$	$\mathbf {0.834} \pm \mathbf {0.094}$	$0.428 \pm 0.092$	$0.765 \pm 0.177$	$0.368 \pm 0.381$
Precision	$\mathbf {0.861} \pm \mathbf {0.115}$	$0.425 \pm 0.119$	$0.751 \pm 0.267$	$0.305 \pm 0.344$
Recall	$0.815 \pm 0.091$	$0.498 \pm 0.144$	$\mathbf {0.894 \pm 0.141}$	$0.548 \pm 0.498$
Dataset	Random walk—linear			
Length (M)	51	11	141	$161.01 \pm 80.38$
$\mathrm{F}_1$	$\mathbf {0.977} \pm \mathbf {0.018}$	$0.024 \pm 0.024$	$0.269 \pm 0.393$	$0.007 \pm 0.034$
Precision	$\mathbf {0.999} \pm \mathbf {0.004}$	$0.127 \pm 0.092$	$0.284 \pm 0.425$	$0.006 \pm 0.030$
Recall	$\mathbf {0.956} \pm \mathbf {0.033}$	$0.014 \pm 0.015$	$0.266 \pm 0.387$	$0.015 \pm 0.104$

The $\mathrm{F}_1$ scores reached their maxima when the expected anomaly length parameters were close to the mean of the actual anomaly lengths for all algorithms and for all detectable cases when the $\mathrm{F}_1$ score showed significant peaks (Table 2).

As we have seen, the variable and unknown length of the anomalies had a significant effect on the detection performance of all methods, but especially LOF and brute force discord detection. Senin et al.^20,46^ extended the discord detection method to overcome the problem of predefined anomaly length and to allow the algorithm to find the length of the anomalies. Thus, we have tested Senin’s algorithm on our test data series and included the anomaly lengths found by this algorithm as well as the performance measures into the comparison in Table 2. While the mean estimated anomaly lengths were not far from the mean of the actual lengths, the performance of this algorithm lags well behind all three previously tested ones on all four types of test data series.

We have identified several factors, which could explain the different detection patterns of different algorithms. Table S1 shows that the tent map and the tachycardia produce lower density, thus more dispersed points in the state space, presumably making them more detectable by the LOF. In contrast, linear segments resulted in a similar density of points to the normal logistic activity or a higher density of points compared to the random walk background. Detrending via differentiation of the logarithm was applied as a preprocessing step in the latter case, making the data series stationary and drastically increasing the state space density of the anomaly.

LOF relies solely on the local density, thus it only counts the low-density sets as outliers. In contrast, as discord detection method identifies anomalies based on the distances in the state space, it was able to detect linear anomaly on chaotic background, tent-map anomaly on log-map data series, and tachycardia on the simulated ECG data, but failed on the detection of the linear anomaly on random walk background. The state-space points belonging to the well-detected anomalies are truly farther from the points in the manifolds of the background dynamics (Fig. 1A-C). In contrast, after discrete-time derivation of logarithms, the points belonging to a linear anomaly are placed near the center of the background distribution (Fig. 1D), making them undetectable either for LOF and discord algorithms.

The detection performance of TOF was less affected by the relation between the expected and the actual length of the anomalies in the linear cases. The reason behind this is that each point of the linear segment is a unique state in itself, thus it always falls below the expected maximal anomaly length. In contrast, the tent map and tachycardic anomalies produce short, but stationary segments, which can be less effectively detected if they are longer than the preset expected length.

We can conclude that 1) TOF has reached better performance to detect anomalies in all the investigated cases, 2) there are special types of anomalies that can be detected only by TOF and can be considered unicorns but not outliers or discords.

### TOF detects unicorns only

**Figure 4:**
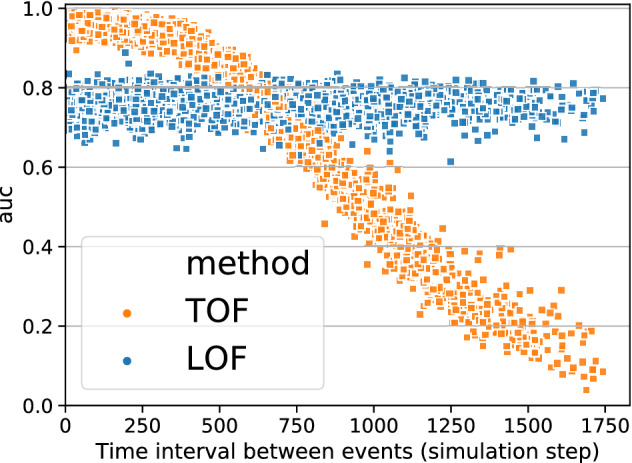
TOF detects unique events only. Detection performance measured by ROC AUC as a function of the minimum Inter-Event Interval (IEI) between two inserted tent-map outlier segments. TOF was able to distinguish outliers from the background very well when IEIs were below 300 steps, and the two events can be considered one. However, the detection performance of TOF decreased for higher IEIs. In contrast, LOF’s peak performance was lower, but independent of the IEI.

To show that TOF enables detection of only unique events, additional simulations were carried out, where two, instead of one, tent-map outlier segments were inserted into the logistic map simulations. We detected outliers by TOF and LOF and subsequently, ROC AUC values were analyzed as a function of the Inter-Event Interval (IEI, Fig. 4) of the outlier segments. LOF performed independent of IEI, but TOF’s performance showed strong IEI-dependence. The highest TOF ROC AUC values were found at small IEI-s and AUC was decreasing with higher IEI. Also, the variance of ROC AUC values was increasing with IEI. This result showed that the TOF algorithm can detect only unique events: if two outlier events are close enough to each other, they can be considered as one unique event together. In this case, TOF can detect it with higher precision, compared to LOF. However, if they are farther away than the time limit determined by the detection threshold, then the detection performance decreases rapidly.

The results also showed that anomalies can be found by TOF only if they are alone, a second appearance decreases the detection rate significantly.

### Application examples on real-world data series

#### Detecting apnea event on ECG time series

To demonstrate that the TOF method can reveal unicorns in real-world data, we have chosen data series where the existence and the position of the unique event are already known.

We applied TOF to ECG measurements from the MIT-BIH Polysomnographic Database’s^47,48^ to detect an apnea event. Multichannel recordings were taken on 250 Hz sampling frequency, and the ECG and respiratory signal of the first recording was selected for further analysis ($n=40{,}000$ data points 1600 s.

While the respiratory signal clearly showed the apnea, there were no observable changes on the parallel ECG signal.

**Figure 5:**
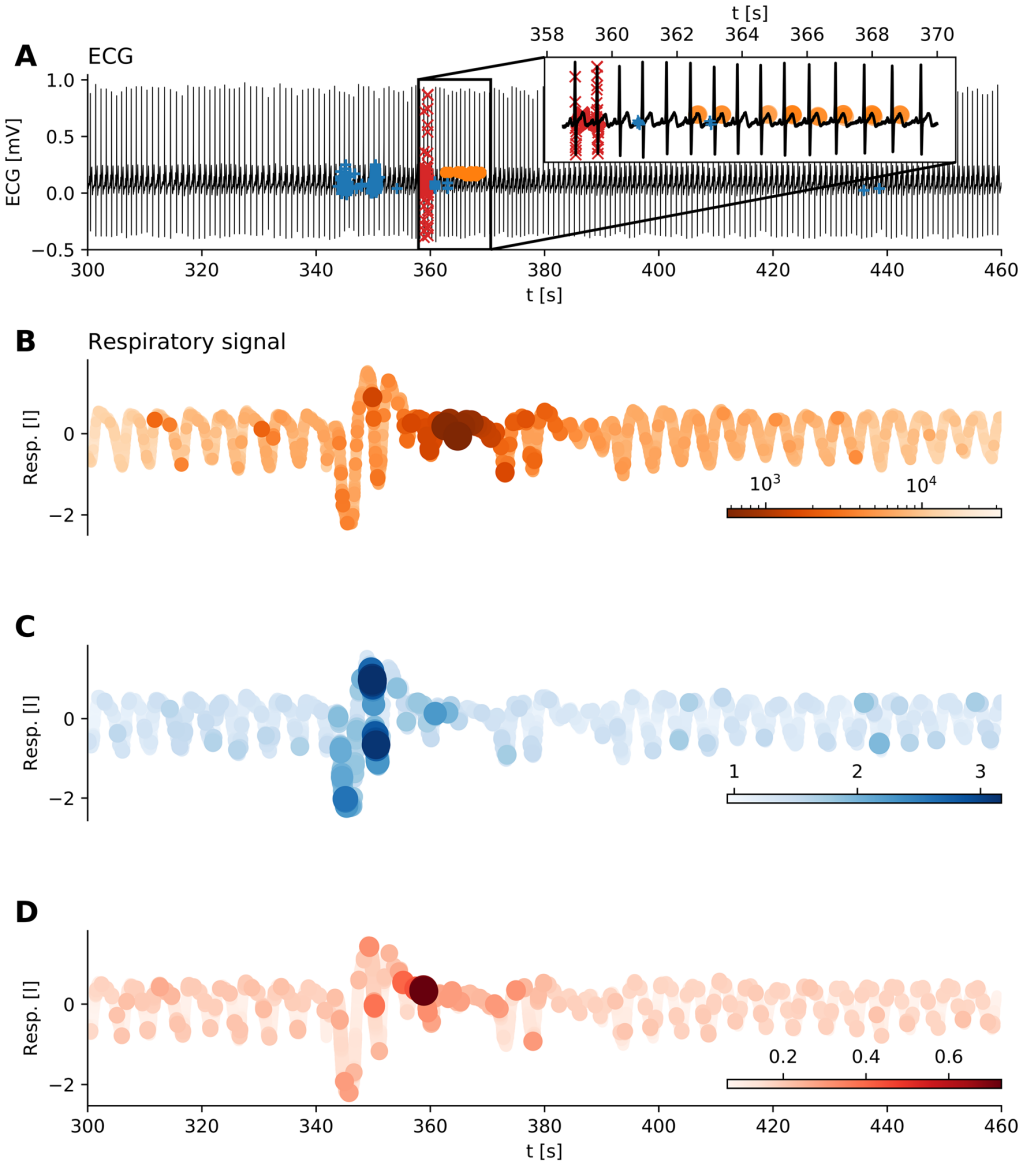
Detecting apnea with arousal on ECG. (**A**) ECG time series with unique events detected by TOF (orange dots, $E=3, \tau =0.02 \,\mathrm{s}, k=11, M=5 \,\mathrm{s}$), outliers detected by LOF (blue + signs, $E=7,\tau =0.02 \,\mathrm{s}, k=100$, threshold $=0.5 \%$) and the top discord (red x signs, M=5 s). The inset shows the more detailed pattern of detections: unique behavior mainly appears on the T waves. (**B**–**D**) Breathing air-flow time series parallel to the above ECG recording, colored according to the scores of the three anomaly methods. The anomaly starts with a period of irregular breathing at 340 s, followed by the apnea when breathing almost stops (350–370 s). After this anomalous period, arousal restores the normal breathing. (**B**) Airflow is colored according to the TOF score at each sample. Low values (darker colors) mark the anomaly corresponding to the period of apnea. (**C**) Air-flow time series with coloring corresponds to the LOF score at each sample. Higher LOF values mark the outliers. LOF finds irregular breathing preceding the apnea. (**D**) Airflow time series colored according to the matrix profile values by the discord. Discord detection algorithm finds the point of transition from irregular breathing to the apnea.

We applied time delay embedding with $E_{\mathrm{TOF}}=3$, $E_{\mathrm{LOF}}=7$ and $\tau =0.02\,\mathrm{s}$ according to the first zero crossing of the autocorrelation function (Fig. S9). TOF successfully detected apnea events in ECG time series; interestingly, the unique behaviour was found mostly during T waves when the breathing activity was almost shut down (Fig. 5, $k=11$, $M=5\,\mathrm{s}$). In contrast, LOF was sensitive to the increased and irregular breathing before apnea ($k=200$, threshold$=0.5{\,\%}$), while the top discord ($M=5\,\mathrm{s}$) were found at the transient between the irregular breathing and the apnea. This example shows that our new method could be useful for biomedical signal processing and sensor data analysis.

#### Detecting gravitational waves

**Figure 6:**
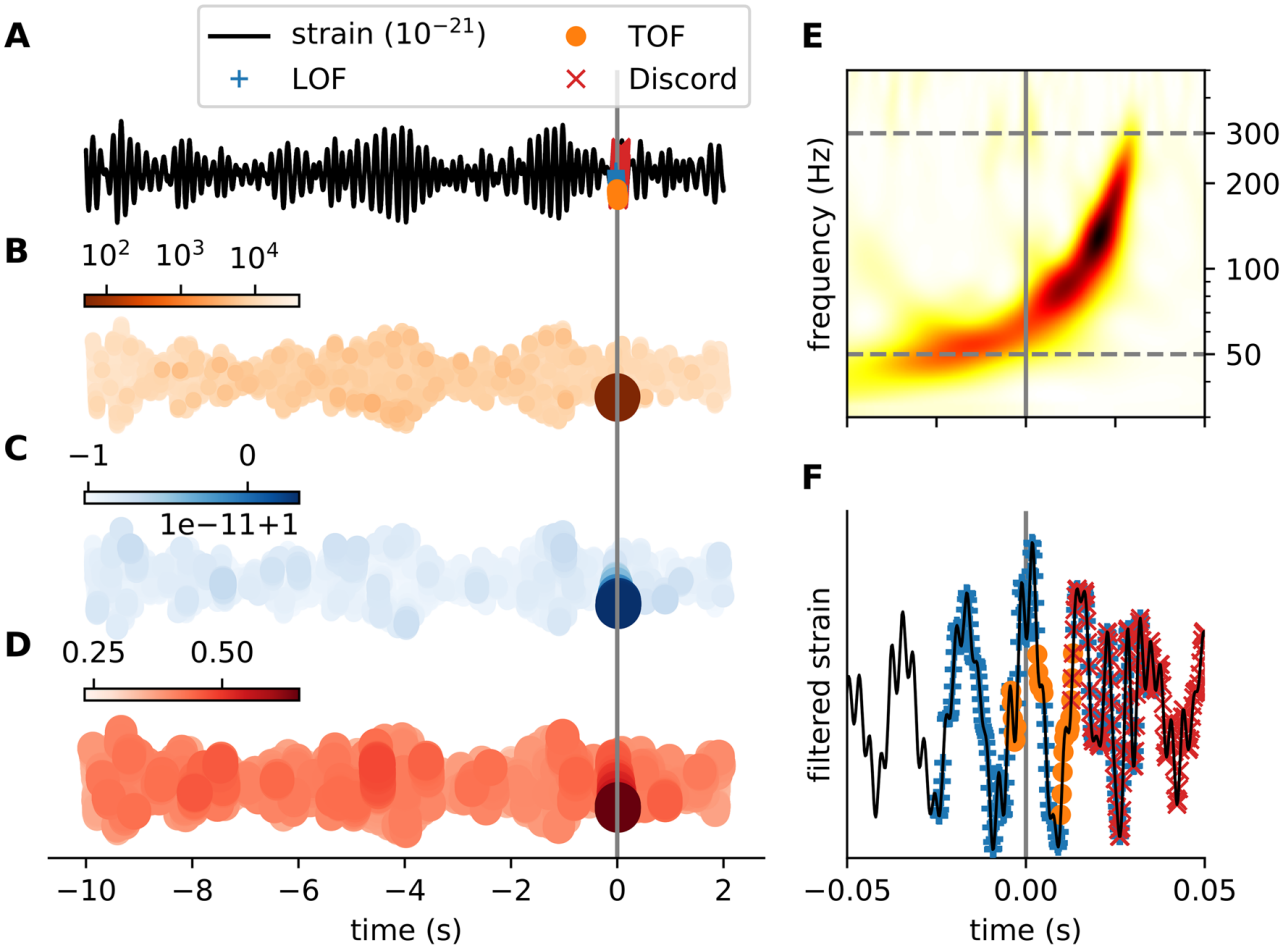
Detection of the GW150914 event on LIGO open data with TOF and LOF and discord. (**A**) Strain time series (black) from Hanford detector around GW150914 event (grey vertical line) with TOF (orange dots), LOF (blue plus) and discord (red x) detections. TOF score values (**B**), LOF scores (**C**) and matrix profile scores (**D**) are mapped to the time series (orange, blue and red colors respectively), the strongest colors show the detected event around 0 s. (**E**) The Q-transform of the event shows a rapidly increasing frequency bump in the power spectra right before the merger event (grey). The grey horizontal dashed lines show the lower (50 Hz) and upper (300 Hz) cutoff frequencies of the bandpass filter, which was applied on the time series as a preprocessing step before anomaly detection. (**F**) Filtered strain data at 0.1 s neighborhood around the event. TOF, LOF, and discord detection algorithms detected the merger event with different sensitivity. LOF detected more points of the event, while TOF found the period which has the highest power in the power spectra, and a discord was detected at the end of the event. ($E_{\mathrm{TOF}}=6$, $\tau _{\mathrm{TOF}}=1.953$ ms, $k_{\mathrm{TOF}}=12$, $M_{\mathrm{TOF}}=146.484$ ms, $w=7$; $E_{\mathrm{LOF}}=11$, $\tau _{\mathrm{LOF}}=1.953$ ms, $k_{\mathrm{LOF}}=100$, threshold$=0.5$%, $M_{discord}=146.484\,{\text{ms}}$).

As a second example of real-world datasets with known unique events, we analyzed gravitational wave detector time series around the GW150914 merger event^18^ (Fig. 6). The LIGO Hanford detector’s signal (4096 Hz) was downloaded from the GWOSC database^49^. A 12 s long segment of strain data around the GW150914 merger event was selected for further analysis. As a preprocessing step, the signal was bandpass-filtered (50–300 Hz). Time delay embedding was carried out with embedding delay of 8 time-steps (1.953 ms) and embedding dimension of $E=6$ and $E=11$ for TOF and LOF respectively. The neighbor parameter was set to $k=12$, for TOF and $k=100$ for LOF. The length of the event was set to $M=146.484\,{\text{ms}}$ for TOF and discord detection and correspondingly, the threshold to $0.5 \%$ for LOF (Fig. S10).

All three algorithms detected the merger event, albeit with some differences. LOF found the whole period, while TOF selectively detected the period when the chirp of the spiraling black holes was the loudest. Interestingly, the top discord found the end of the event (Fig. 6B-D).

To investigate the performance of TOF on detecting noise bursts called blip in LIGO detector data series, we applied the algorithm on the Gravity Spy^50^ blip data series downloaded from the GWOSC database^49^ (Fig. S7). We determined the value of the optimal threshold on the training set ($N=128$), then measured precision, F$_1$ score, recall, and block-recall metrics on the test set ($N=29$). We set the threshold value by the maximum precision ($M=36$, Fig. S7A). TOF reached high precision (1), low $\hbox {F}_1$ score, low recall and high block-recall (0.9) values (Fig. S7B) on the test set. The high precision shows that the detected anomaly is likely to be a real blip and the high block recall (hit rate) implies that TOF found blips in the majority of the sample time series.

#### London InterBank Offer Rate dataset

Our final real-world example is the application of TOF, LOF, and discord detection algorithms on the London InterBank Offer Rate (LIBOR) dataset. In this case, we have no exact a priori knowledge about the appearance of unique events, but we assumed that unique states found by the TOF algorithm may have unique economic characteristics.

**Figure 7:**
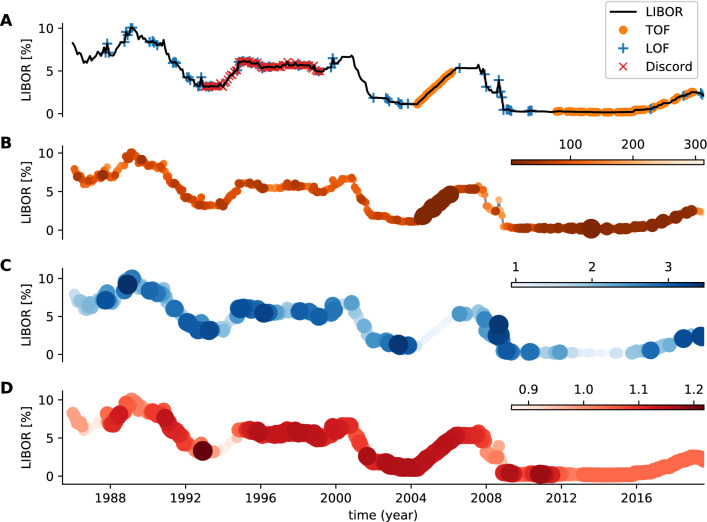
Analysis of LIBOR dataset. The detections were run on the temporal derivative of the LIBOR time series. (**A**) time-series with detections. (**B**) TOF score values. (**C**) LOF score values. (**D**) Matrix profile scores by the discord detection algorithm. TOF detected two rising periods: the first between 2005 and 2007 and a second, started in 2012 and lasts until now. While both periods exhibit unique dynamics, they differ from each other as well.

As a preprocessing step, discrete time derivative was calculated to eliminate global trends, then we applied TOF ($E=3, \tau =1, k=5, M=30$ month) and LOF ($E=3, \tau =1, k=30$, $\hbox {threshold}=18.86\,\%$) on the derivative (Figs. S11-S12). TOF found the uprising period prior to the 2008 crisis and the slowly rising period from 2012 onwards as outlier segments. LOF detected several points, but no informative pattern emerged from the detections (Fig. 7). Also, Discord detected a period between 1993 and 1999, with no obvious characteristic.

While in this case the ground-truth was not known, the two periods highlighted by TOF show specific patterns of monotonous growth. Moreover, the fact that both of the two periods were detected by TOF shows that both dynamics are unique, therefore different from each other.

## Discussion

In this paper we introduced a new concept of anomalous event called unicorn; unicorns are the unique states of the system, which were visited only once. A new anomaly concept can be valid only if a proper detection algorithm is provided: we have defined the Temporal Outlier Factor to quantify the uniqueness of a state. We demonstrated that TOF is a model-free, non-parametric, domain-independent anomaly detection tool, which can detect unicorns.

TOF measures the temporal dispersion of state-space neighbors for each point. If state-space neighbors are temporal neighbors as well, then the system has never returned to that state, therefore it is a unique event. ie. a unicorn.

The unicorns are not just outliers in the usual sense, they are conceptually different. As an example of their inherently different behavior, one can consider a simple linear data series: All of the points of this series are unique events; they are only visited once and the system never returned to either one of them. Whilst this property may seem counter-intuitive, it ensures that our algorithm finds unique events regardless of their other properties, such as amplitude or frequency. This example also shows that the occurrences of unique events are not necessarily rare: actually, all the points of a time series can be unique. This property clearly differs from other anomaly concepts: most of them assume that there is a normal background behavior that generates the majority of the measurements and outliers form only a small minority.

Keogh’s discord detection algorithm^19^ differs from our method in an important aspect: Keogh’s algorithm finds one, or other predefined number of anomalies on any dataset. Thus Keogh’s algorithm can not be used to distinguish, whether there are any anomalies on the data or not, it will always find at least one. This property makes it inappropriate in many real-world applications since usually, we do not know if there are any anomalies on the actual dataset or not. In contrast, our algorithm can return any number of anomalies, including zero.

Detection performance comparison of TOF, LOF, and two discord detection algorithms on different simulated datasets highlighted the conceptual difference between the traditional outliers and the unique events as well. As our simulations showed, TOF with the same parameter settings was able to find both higher and lower density anomalies, based on the sole property that they were unique events. The algorithm has a very low false detection rate, but not all the outlier points were found or not all the points of the event were unique. As an example, QRS waves of ECG simulations do not appear to be different from normal waves, hence the algorithms did not find them.

Of course, our aim was not to compete with those specific algorithms that have been developed to detect sleep apnea events from ECG signal^51^. Most of the methods extract and classify specific features of the R-R interval series called heart rate variability (HRV). It was shown, that sympathetic activation during apnea episodes leaves its mark on HRV^52^, its spectral components, sample entropy^53^ or correlation dimension^54^. Song et al.^55^ used discriminative Markov-chain models to classify HRV signals and reached $97\%$ precision for per-recording classification.

While ECG analysis mostly concentrates on the temporal relations of the identified wave components, here we apply the detection methods to the continuous ECG data. Previously, it was shown that apnea is associated with morphological changes of the P waves and the QRS complex in the ECG signal^51,56,57^.

Interestingly, TOF marked mainly the T waves of the heart cycle as anomalous points. T waves are signs of ventricular repolarization and are known to be largely variable, thus they are often omitted from the ECG analysis. This example showed that they can carry relevant information as well.

The already identified gravitational wave GW150914 event was used to demonstrate the ability of our method to find another type of anomaly without prior knowledge about it.

Clearly, specific model-based algorithms (such as matched filter methods^58^) or unmodelled algorithms that were originally used to recognize gravitational waves, such as coherent Wave Bursts, omicron-LALInference-Bursts, and BayesWave are much more sensitive to the actual waveforms generated by the merger of black holes or neutron stars than our TOF method^59^. The unmodelled methods have only two basic assumptions: first, that the gravitational wave background (unlike ECG signal) is basically silent, thus detectors measure only Gaussian noise in the absence of an event. Thus, any increase in the observed wave-power needs to be detected and classified. Second, an increase in the coherent power between the far located detectors is the hallmark of candidate events of astrophysical origin. The detectors should observe similar waveforms with phase difference corresponding to the waves traveling with light-speed between them. In contrast, increased power in only one of the detectors should have a terrestrial origin and these are called glitches. After the unmodelled detection of candidate waveforms, more specific knowledge about the possible waveforms can be incorporated into the analysis pipeline, such as analyzing time evolution of the central frequency of the signal, or comparison of the waveform to the model database, containing simulated waveforms generated by merger events. Model-free methods can detect events with unpredicted waveforms and may help to find glitches. The presence of different types of glitches significantly increases the noise level and decreases the useful data length of detectors, thus limiting its sensitivity.

In contrast to apnea and gravitational wave detection, the nature of anomalies is much less known in the economical context. Most of these anomaly detection methods concentrate on fraud detection of transaction or network traffic records and utilize clustering techniques to distinguish normal and fraudulent behaviors^60^.

Whilst LOF showed no specific detection pattern, TOF detected two rising periods on the temporal derivative of the USD LIBOR dataset: one preceding the 2008 crisis and another one from 2012 onwards. Both detected periods showed unique dynamics: the large fluctuations are replaced by constant rising during these periods, the dynamics are ’frozen’. Note, that the rising speeds differ in the two periods. The period between 2005-2007 can be considered unique in many ways; not only was there an upswing of the global market, but investigations revealed that several banks colluded in manipulation and rigging of LIBOR rates in what came to be known as the infamous LIBOR scandal^61^. Note, that this was not the only case when LIBOR was manipulated: During the economic breakdown in 2008 the Barclys Bank submitted artificially low rates to show healthier appearance^62–64^. As a consequence of these scandals, significant reorganization took place in controlling LIBOR calculation, starting from 2012.

To sum it up, gravitational waves of the merger black-holes on the filtered dataset formed a traditional outlier which was well detectable by all the TOF, the LOF, and the discord detection algorithms, while LIBOR exhibited longer periods of unique events only detectable by TOF. Apnea generated a mixed event on ECG; the period of irregular breathing formed outliers detectable by LOF, while the period of failed respiration generated a unique event detectable only by the TOF. Meanwhile, the top discord was found at the transitory period between the two states.

Comparing TOF, LOF, and discord detection algorithms proved that temporal scoring has advantageous properties and adds a new aspect to anomaly detection. One advantage of TOF can be experienced when it comes to threshold selection. Since the TOF score has time dimension, an actual threshold value means the maximal expected length of the event to be found. Also, on the flipside the neighborhood size *k* parameter sets the minimal event length. Because of these properties, domain knowledge about possible event lengths renders threshold selection a simple task.

While TOF and LOF have similar computational complexity ($O(k n \log (n))$), the smaller embedding dimensions and neighborhood sizes make TOF computations faster and less memory hungry. While the brute force discord detection algorithm has $O(k n^2 \log {n})$ complexity^19^, the running time of discord detection has been significantly accelerated by the SAX approximation^19^ and latter the DRAG algorithm, which is essentially linear in the length of the time series^65^. However, our results may indicate that the SAX approximation has seriously limited the precision of Senin’s algorithm.

To measure the running time empirically, we applied TOF algorithm on random noise from $10^2-10^6$ sample size, 15 instances each ($d=3$, $\tau =1$, $k=4$). The runtime on the longest tested $10^6$ points long dataset was $15,144\pm 0.351$ secs (Fig. S4) on a laptop powered by Intel® Core$^{\mathrm{TM}}$ i5-8265U. The fitted exponent of the scaling was 1.3. Based on these results, we have estimated that if memory issues could be solved, running a unicorn search on the whole 3 months length of the LIGO O1 data downsampled to 4096Hz would take 124 days on a single CPU (8 threads). A search through one week of ECG data would take 3 hours. As calculations on the ECG data are much shorter than the recording length; online processing is feasible as well.

Time indices of k nearest neighbors have been previously utilized differently in nonlinear time series analysis to diagnose nonstationary time series^24,33,66^, measure intrinsic dimensionality of system’s attractors^34–36^, monitor changes in dynamics^37^ and even for fault detection^38^. Rieke et al. ^33,66^ utilized very resembling statistics to TOF: the average absolute temporal distances of k nearest neighbors from the points. However, they analyzed the distribution of temporal distances to determine nonstationarity and did not interpret the resulting distance scores locally. Gao & Hu and Martinez-Rego et al.^38^ used recurrence times to monitor dynamical changes in time series locally, but these statistics are not specialized for detecting extremely rare unique events. TOF utilizes the temporal distance of k nearest neighbors at each point, thus providing a locally interpretable outlier score, which takes small values when the system visits an undiscovered territory of state-space for a short time period.

The minimal detectable event length might be the strongest limitation of the TOF method. We have shown that the TOF method has a lower bound on the detectable event length ($\Theta _{min}$), which depends on the number of neighbors (*k*) used in the TOF calculations. This means that TOF is not well suited to detect point-outliers, which are easily detectable by many traditional outlier detection methods.

Furthermore, the shorter the analyzed time series and the smaller *k* is used, the higher the chance that the background random or chaotic dynamics spontaneously produce a unique event. Smaller *k* results in higher fluctuations of the baseline TOF values, which makes the algorithm prone to produce false-positive detections.

A further limitation arises from the difficulty of finding optimal parameters for the time delay embedding: the time delay $\tau$ and the embedding dimension *E*. Figure S5 shows the sensitivity of the $F_1$ score to the time delay embedding parameters and the relation between the used and the optimal parameter pairs. This post hoc evaluation, which can be done for simulations but not in a real-life data showed, that our general parameter setting ($E=3$, $\tau =1$) used during the tests was suboptimal for the simulated ECG-tachycardia dataset. The optimal parameter settings ($E=7$, $\tau =6$) would have resulted in 0.94 as the maximal $F_1$ score instead of 0.83, shown in Table 2).

The model-free nature of these algorithms can be an advantage and a limitation at the same time. The specific detection algorithms, which are designed on purpose and use specifically a priori knowledge about the target pattern to be detected, can be much more effective than a model-free algorithm. Model-free methods are preferred when the nature of the anomaly is unknown. Consequently, detecting a unicorn tells us that the detected state of the system is unique and differs from all other observed states, but it is not often obvious in what sense; posthoc analysis or domain experts are needed to interpret the results.

Preprocessing can eliminate information from the data series, thus can filter out aspects considered uninteresting. For example, we have seen that a strong global trend on data can make all the points unique. By detrending the data, as done on random walk and LIBOR datasets, we defined that these points should not be considered unique solely based on this feature. Similarly, band-pass filtering of gravitational wave data defines that states should not be considered unique based on the out-of-frequency-range waveforms.

Future directions to develop TOF would be to form a model which is able to represent uncertainty over detections by creating temporal outlier probabilities just like Local Outlier Probabilities^67^ created from LOF. Moreover, an interesting possibility would be to make TOF applicable also on different classes of data, such as multi-channel data or point processes, like spike-trains, network traffic time-stamps or earthquake dates.

## Supplementary information

Supplementary Information.
