# Supplementary material for: Model-free detection of unique events in time series
Source: Sci Rep. 2022 Jan 7;12:227. doi: 10.1038/s41598-021-03526-y (PMC8742065; doi:10.1038/s41598-021-03526-y)
Supplement: Supplementary file 1 — Supplementary Information. [file 41598_2021_3526_MOESM1_ESM.pdf]

Supplementary Information for Model-free detection of unique events in time series

Zsigmond Benkő[1,2], Tamás Bábel[1], and Zoltán Somogyvári[1,*]

[1]Wigner Research Centre for Physics, Department of Computational Sciences, Budapest, H-1121, Hungary
[2]Semmelweis University, János Szentágothai Doctoral School of Neurosciences, Üllői road 26., Budapest, H-1085, Hungary
[*]somogyvari.zoltan@wigner.hu

ABSTRACT

In this supplementary, we summarise the workflow of the TOF computation, derive analytic results of the TOF values, provide details to the simulations used for testing the detection methods, check the computational complexity of the TOF algorithm by numerical experiments, evaluate the parameter dependence of the detection performance and provide hints on how to chose the embedding parameters for real life data series.

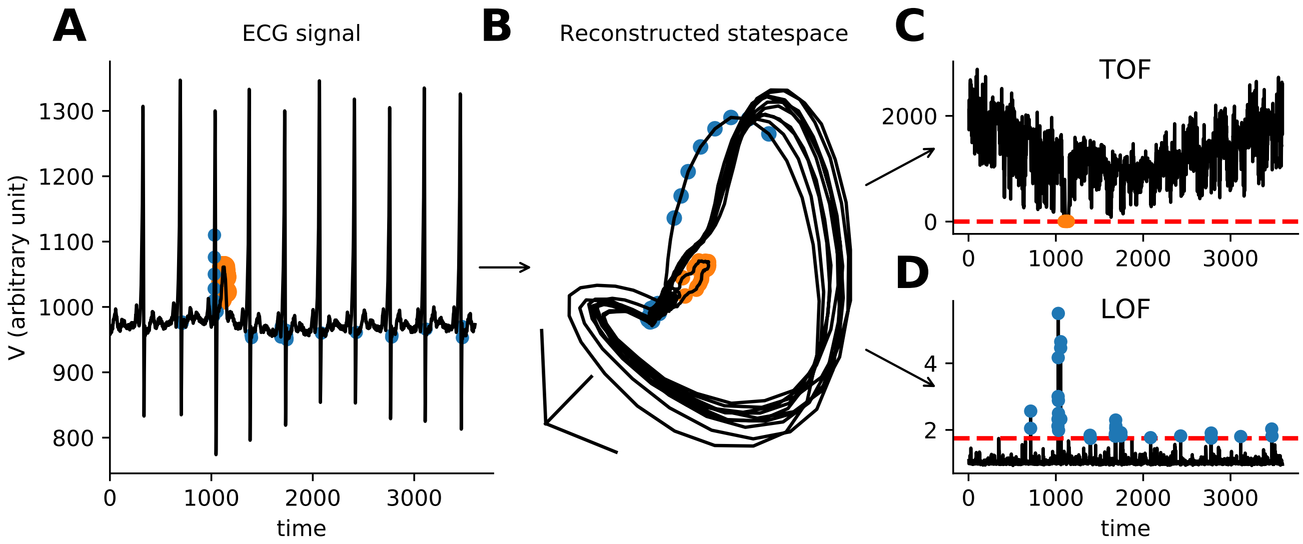

Figure S1. **The worklow for TOF and LOF analysis for time series. A** We start with a time series generated by a dynamical system; orange and blue marks TOF and LOF detections respectively. **B** As a next step of our analysis we apply time delay embedding, then kNN search in the reconstructed state space. **C** and **D** We calculate TOF and LOF scores and apply thresholds on the outlier scores to detect anomalies.

TOF Analysis workflow

The main steps of the TOF analysis are recapitulated here for completeness:

1. Preprocessing and applicability check:

   This step varies from case to case, and depends on the data or on the goals of analysis. Usually it is advisable to make the data stationary. For example, in the case of oscillatory signals, the signal must contain many periods even from the lowest frequency components. If this latter condition does not hold, then Fourier filtering can be applied to get rid of the low frequency components of the signal.

2. Time delay embedding:

   We embed the scalar time series into an E dimensional space with even time delays τ (Fig. 1 **A**):

$$X(t) = [x(t), x(t+\tau), x(t+2\tau), \dots x(t+(E-1)\tau)] \tag{1}$$

   The embedding parameters can be set with prior knowledge of the dynamics or by other optimization methods. Such optimization methods include the first minimum or zerocrossing of the autocorrelation function (for delay selection), the false nearest neighbor method[1,2] or the differential entropy based embedding optimizer that we applied[3]. Figs. S9-S12 illustrates our parameter hunting procedure, where the τ was chosen as the first zero point of the autocorrelation function of the signal or as the first minima, if it does not reach the zero level. The embedding dimension was estimated by finding the embedding dimension where the estimated dimension started to deviate from the embedding dimension. This procedure worked well for dynamical systems (Fig, S9-S10) but not for the LIBOR which is more likely to be generated by a stochastic process. Here, the estimated dimension increased with the embedding dimension without reaching a plateau (Fig. S11). Thus in this case, the embedding dimension was estimated based on the differential entropy (Fig. S12).

3. kNN Neighbor search:

   We search for k-neighborhoods around each datapoint in the statespace using the kDTree algorithm and save the distance and temporal index of neighbors[4].

4. Compute TOF score:

$$\mathrm{TOF}(t) = \sqrt[q]{\frac{\sum_{i=1}^{k} |t - t_i|^q}{k}}. \tag{2}$$

   Where t is the time index of the sample point ($X(t)$) and t_i is the time index of the i-th nearest neighbor in reconstructed state-space. Where $q \in \mathscr{R}^+$, in our case we use $q = 2$.

5. Apply a threshold θ on TOF score to detect unicorns (Fig. S1 **C**):

   The threshold can be established by prior knowledge, by clustering techniques or supervised learning. The maximum event length parameter (M) determines the level of threshold on TOF score:

$$\theta = \sqrt{\frac{\sum_{i=0}^{k-1} (M - i\Delta t)^2}{k}} \quad \Bigg| \quad k\Delta t \overset{!}{\leq} M \tag{3}$$

   We set the threshold according to prior knowledge about the longest possible occurence of the event. After thresholding, we may apply a padding around detected points with symmetric window length $w = k/2$, since the k parameter sets the minimal length of the detectable events.

We implemented these steps in the python programming language (python3), the software is available at https://github.com/phrenico/uniqed.

The code builds on standard scientific python modules, i. e. the neighborhood search is established by the kd-tree algorithm of the scipy package[5]. Embedding parameter optimization was carried out by custom python scripts.

Furthermore, we used the scikit-learn package[6] to calculate LOF. We implemented the brute-force discord discovery algorithm[7] by custom python and scilab scripts and we used the R implementation of Rare Rule Anomaly (RRA)[8,9] (Senin) discord discovery algorithm on all simulated datasets.

Mean and variance for $q = 1$

The mean and the variance of TOF can be computed for uncorrelated noise in the continuous-time limit, where the typical properties of the metrics can be introduced. The expectation of the first neighbor is easy to compute (Eq. 4), if we take the probability density function ($p(\tau)$) as uniform; this is the assumption of white noise. Additionally, the pdf is independent of the rank of the neighbor (k), and thus the mean is the same for all neighborhood sizes. By the previous assumptions, the mean is simply a quadratic expression:

$$\langle TOF_{q=1} \rangle \quad = \int_0^T |t - \tau| \, p(\tau) \, \mathrm{d}\tau \; = \; \frac{1}{T} \int_0^T |t - \tau| \, \mathrm{d}\tau \; = \; \frac{t^2}{T} - t + \frac{T}{2} \tag{4}$$

with the method of moments, we calculate the variance for $k = 1$:

$$\langle TOF^2_{q=1} \rangle \quad = \int_0^T (t-\tau)^2\, p(\tau)\,\mathrm{d}\tau \;=\; \frac{1}{T}\int_0^T (t-\tau)^2\,\mathrm{d}\tau \;=\; t^2 - tT + \frac{T^2}{3} \tag{5}$$

$$\sigma^2_{q=1} \quad = \langle TOF^2_{q=1} \rangle - \langle TOF_{q=1} \rangle^2 \;=\; -\frac{t^4}{T^2} + \frac{2t^3}{T} - t^2 + \frac{T^2}{12} \tag{6}$$

if we have k neighbors, then the variance is reduced by a $1/k$ factor:

$$\sigma^2_{q=1,k} \quad = \langle TOF^2_{q=1} \rangle - \langle TOF_{q=1} \rangle^2 \;=\; \frac{1}{k}\left(-\frac{t^4}{T^2} + \frac{2t^3}{T} - t^2 + \frac{T^2}{12}\right) \tag{7}$$

To test whether these theoretical arguments fit to data, we simulated random noise time series ($n = 100, T = 1000$) and computed the mean TOF score and standard deviation (Fig. 2). We found, that theoretical formulas described the behaviour of TOF perfectly.

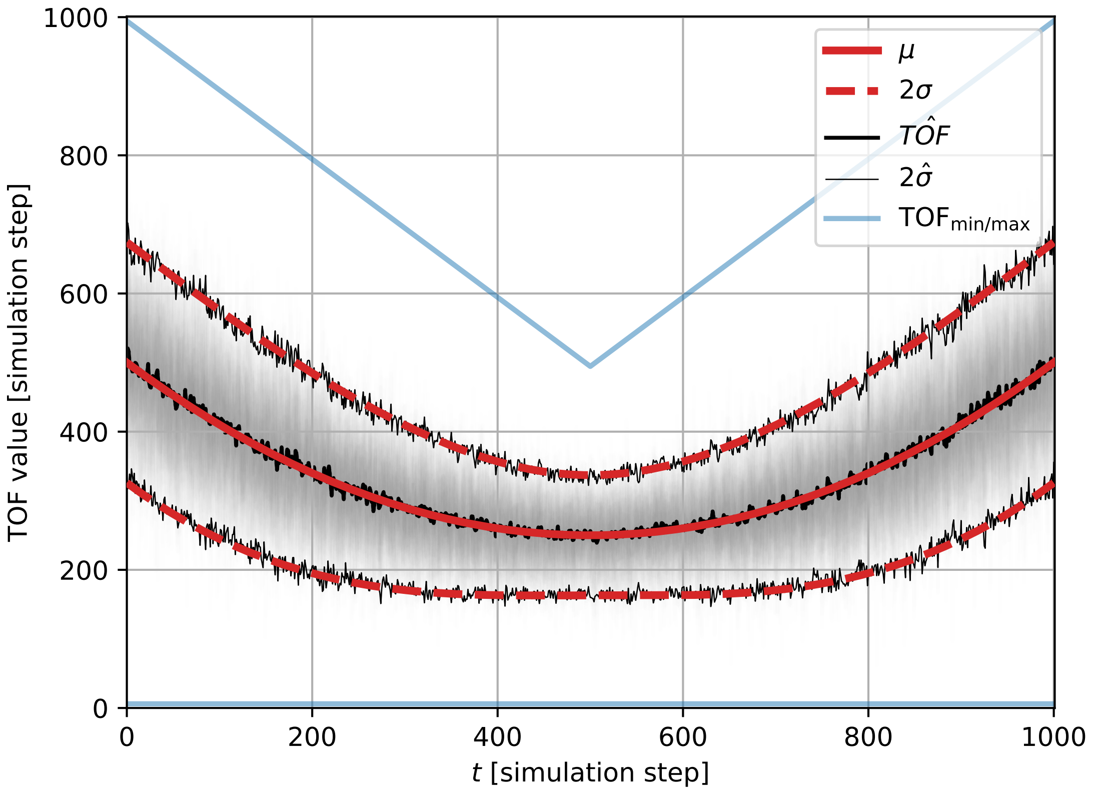

Figure S2. Properties of TOF for white noise data: theory and simulations. The expectation of TOF is computed as a function of temporal position in the time series ($q = 1$, thick red line), also the standard deviation was calculated (dashed red line). The average (thick black line) and standard deviation (thin black line) of $n = 100$ instances (grey shading). The minimal and maximal possible TOF vales are also charted (blue lines).

Mean and variance for $q = 2$

The exact statistics is hard to calculate, when the value of the q exponent is not equal to one. Here we compute a vague approximation for $q = 2$ (Fig. 3). By computing the mean and variance for TOF squared, and taking the squareroot of these values can get a feeling about the properties of $\mathrm{TOF}_{q=2}$ respectively.

$$\left\langle \mathrm{TOF}^2_{\mathrm{noise},q=2} \right\rangle = \int_0^T (t-\tau)^2\, p(\tau)\,\mathrm{d}\tau \;=\; \frac{1}{T}\int_0^T (t-\tau)^2\,\mathrm{d}\tau \;=\; t^2 - tT + \frac{T^2}{3} \tag{8}$$

the second moment is as follows:

$$\left\langle \mathrm{TOF}^4_{\mathrm{noise},q=2} \right\rangle = \int_0^T (t-\tau)^4 \, p(\tau)\, \mathrm{d}\tau \; = \; \frac{1}{T} \int_0^T (t-\tau)^4 \, \mathrm{d}\tau \; = \; \frac{t^5+(T-t)^5}{5T} \tag{9}$$

Thus using the method of moments we can get the variance of the $TOF^2_{q=2}$:

$$\mathrm{Var}\left(\mathrm{TOF}^2_{\mathrm{noise},q=2}\right) \; = \; \frac{t^5+(T-t)^5}{5T} - \left(t^2 - tT + \frac{T^2}{3}\right)^2 \tag{10}$$

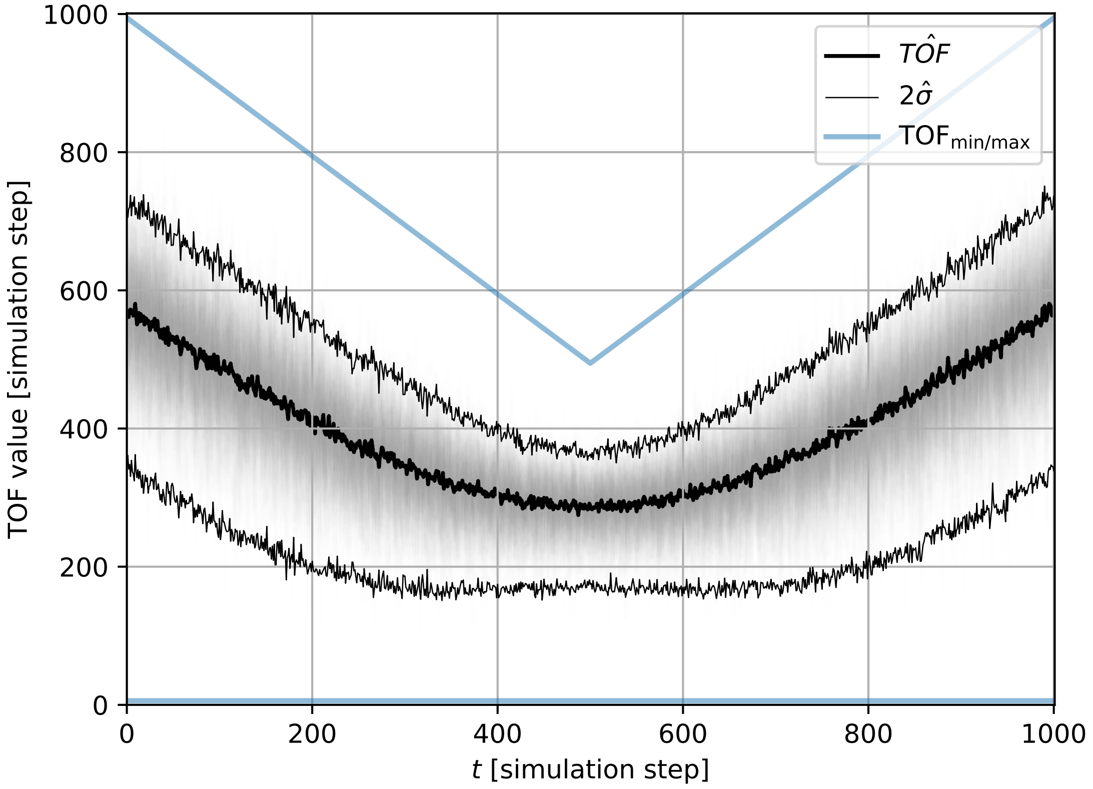

Figure S3. Properties of TOF for white noise data 2: simulations. The baseline of TOF with $q = 2$. The average (thick black line) and standard deviation (thin black line) of $n = 100$ instances (grey shading).

Generation of simulated datasets

Simulated logistic map and stochastical datasets

We simulated 4 systems: logistic map with linear tent map outlier segment, logistic map with linear outlier segment, simulated ECG data with tachycardia outlier segment and random walk with linear outlier segment. The first three datasets stem from deterministic dynamics, whereas the last simulated dataset has stochastic nature.

We generated 100 time series from each type, the length and the position of outlier segments were determined randomly in each case.

Logistic map with tent-map anomaly

100 instances of logistic map data-series were simulated ($N = 2000$) with one randomly (uniform) inserted outlier period in each dataset. The length of outlier periods was randomly chosen with length between $2-200$. The basic dynamics in normal conditions were governed by the update rule:

$$x_{t+1} = rx_t(1-x_t) \tag{11}$$

where $r = 3.9$. The equation was changed during anomaly periods:

$$x_{t+1} = 1.59 - 2.15 \times |x_t - 0.7| - 0.9 \times x_t \tag{12}$$

To make sure that the time series was bounded in the $I = [0,1]$ interval, reflective boundary condition is used, restricting the time series to the desired interval I.

Logistic map with linear anomaly

The background generation process exhibited the logistic dynamics (Eq. 11), while the anomaly can be described by linear time dependence:

$$x_{t+1} = a * x_t + x_t \tag{13}$$

Here we used $a = \pm 0.001$, where the sign of the slope is positive by default and changes when the border of the $(0,1)$ domain is reached ensuring reflective boundary condition.

Random walk data with linear anomaly

We simulated 100 instances of multiplicative random walks with 2-200 timestep long linear outlier-insets. The generation procedure was as follows:

1. Generate w_i random numbers from a normal distribution with $\mu = 0.001$ and $sigma = 0.01$
2. Transform w_i to get the multiplicative random walk data as follows: $x_i = \prod_{j=1}^{i}(1 + w_j)$
3. Draw the length (L) and position of outlier-section from discrete uniform distributions between $2 - 200$ and $1 - (N - L)$ respectively.
4. Use linear interpolation between the section-endpoint values.

Simulated ECG datasets with tachyarrhythmic segments

We generated artificial ECG data series according to the model of Ryzhii and Ryzhii[10]. The pacemakers of the heart: the sinoatrial node (SA), the atroventricluar node (AV) and the His-Purkinje system (HP) are simulated by van der Pol equations:

$$SN \begin{cases} \dot{x}_1 &= y_1 \\ \dot{y}_1 &= -a_1 y_1 (x_1 - u_{11})(x_1 - u_{12}) \\ & \quad - f_1 x_1 (x_1 + d_1)(x_1 + e_1) \end{cases} \tag{14}$$

$$AV \begin{cases} \dot{x}_2 &= y_2 \\ \dot{y}_2 &= -a_2 y_2 (x_2 - u_{21})(x_2 - u_{22}) \\ & \quad - f_2 x_2 (x_2 + d_2)(x_2 + e_2) \\ & \quad + K_{SA-AV}(y_1^{\tau_{SA-AV}} - y_2) \end{cases} \tag{15}$$

$$HP \begin{cases} \dot{x}_3 &= y_3 \\ \dot{y}_3 &= -a_3 y_3 (x_3 - u_{31})(x_1 - u_{32}) \\ & \quad - f_3 x_3 (x_3 + d_3)(x_3 + e_3) \\ & \quad + K_{AV-HP}(y_2^{\tau_{AV-HP}} - y_3) \end{cases} \tag{16}$$

where the parameters were set according to Ryzhii[10]: $a_1 = 40$, $a_2 = a_3 = 50$, $u_{11} = u_{21} = u_{31} = 0.83$, $u_{12} = u_{22} = u_{32} = -0.83$, $f_1 = 22$, $f_2 = 8.4$, $f_3 = 1.5$, $d_1 = d_2 = d_3 = 3$, $e_1 = 3.5$, $e_2 = 5$, $e_3 = 12$ and $K_{\mathrm{SA-AV}} = K_{\mathrm{AV-HP}} = f_1$.

The following FitzHugh-Nagumo equations describe the atrial and ventricular muscle depolarization and repolarization responses to pacemaker activity:

$$\text{P wave} \begin{cases} \dot{z}_1 &= k_1(-c_1 z_1 (z_1 - w_{11})(z_1 - w_{12}) \\ & \quad - b_1 v_1 - d_1 v_1 z_1 + I_{\mathrm{AT_{De}}}) \\ \dot{v}_1 &= k_1 h_1 (z_1 - g_1 v_1) \end{cases} \tag{17}$$

$$\text{Ta wave}\begin{cases}\dot{z}_2 &= k_2(-c_2z_2(z_2-w_{21})(z_2-w_{22}) \\ & \qquad -b_2v_2-d_2v_2z_2+I_{\mathrm{AT_{Re}}}) \\ \dot{v}_2 &= k_2h_2(z_2-g_2v_2)\end{cases} \tag{18}$$

$$\text{QRS}\begin{cases}\dot{z}_3 &= k_3(-c_3z_3(z_3-w_{31})(z_2-w_{32}) \\ & \qquad -b_3v_3-d_3v_3z_3+I_{\mathrm{VN_{De}}}) \\ \dot{v}_3 &= k_3h_3(z_3-g_3v_3)\end{cases} \tag{19}$$

$$\text{T wave}\begin{cases}\dot{z}_4 &= k_4(-c_4z_4(z_4-w_{41})(z_4-w_{42}) \\ & \qquad -b_4v_4-d_4v_4z_4+I_{\mathrm{VN_{Re}}}) \\ \dot{v}_4 &= k_4h_4(z_4-g_4v_4)\end{cases} \tag{20}$$

where $k_1 = 2\times10^3$, $k_2 = 4\times10^2$, $k_3 = 10^4$, $k_4 = 2\times10^3$, $c_1 = c_2 = 0.26$, $c_3 = 0.12$, $c_4 = 0.1$ $b_1 = b_2 = b_4 = 0$, $b_3 = 0.015$, $d_1 = d_2 = 0.4$, $d_3 = 0.09$, $d_4 = 0.1$, $h1 = h2 = 0.004$, $h_3 = h_4 = 0.008$, $g_1 = g_2 = g_3 = g_4 = 1$, $w11 = 0.13$, $w_{12} == w_{22} = 1$, $w_{21} = 0.19$, $w_{31} = 0.12$, $w_{32} = 0.11$, $w_{41} = 0.22$, $w_{42} = 0.8$.

The input-currents (I_i) are caused by pacemaker centra.

$$I_{\mathrm{AT_{De}}} = \begin{cases}0 & \text{for} \quad y_1 \le 0 \\ K_{\mathrm{AT_{De}}}y_1 & \text{for} \quad y_1 > 0\end{cases} \tag{21}$$

$$I_{\mathrm{AT_{Re}}} = \begin{cases}-K_{\mathrm{AT_{Re}}}y_1 & \text{for} \quad y_1 \le 0 \\ 0 & \text{for} \quad y_1 > 0\end{cases} \tag{22}$$

$$I_{\mathrm{VN_{De}}} = \begin{cases}0 & \text{for} \quad y_3 \le 0 \\ K_{\mathrm{VN_{De}}}y_3 & \text{for} \quad y_3 > 0\end{cases} \tag{23}$$

$$I_{\mathrm{VN_{Re}}} = \begin{cases}-K_{\mathrm{VN_{Re}}}y_3 & \text{for} \quad y_3 \le 0 \\ 0 & \text{for} \quad y_3 > 0\end{cases} \tag{24}$$

where $K_{\mathrm{AT_{De}}} = 4\times10^{-5}$, $K_{\mathrm{AT_{Re}}} = 4\times10^{-5}$, $K_{\mathrm{VN_{De}}} = 9\times10^{-5}$ and $K_{\mathrm{VN_{Re}}} = 6\times10^{-5}$.

The net ECG signal is given by the weighted sum of muscle depolarization and repolarization responses:

$$ECG = z_0 + z_1 - z_2 + z_3 + z_4 \tag{25}$$

where $z_0 = 0.2$ is a constant offset.

We simulated 100 instances of $t = 100$ seconds long ECG data with base rate parameter chosen from a Gaussian distribution ($f_1 \sim \mathcal{N}(\mu = 22, \sigma = 3)$). We randomly inserted $2-20$ seconds long fast heart-beat segments by adjusting the rate parameter ($f_1 \sim \mathcal{N}(\mu = 82, \sigma = 3)$). The simulations were carried out by the ddeint python package, with simulation time-step $\Delta t = 0.001$ from random initial condition and warmup time of 2 seconds. Also, a $10\times$ rolling-mean downsampling was applied on the data series before analysis.

Generating non-unique anomalies dataset

To show the selectiveness of TOF for the detection of unicorns, we simulated logistic map data with two tent-map outlier segments. The governing equations were the same as in the previous section, but instead of one, we randomly placed two non-overlapping outlier segments into the time series during data generation, ($N = 2000, L = 20-200$).

Analysis steps on simulations

We applied optional preprocessing, and ran TOF, LOF, brute-force discord discovery[7] and Rare Rule Anomaly (RRA)[8] discord discovery algorithms on all simulated datasets.

We applied the same preprocessing on the datasets for all anomaly detection methods on the four datasets. For the logistic map datasets no preprocessing was applied. For the simulated ECG data we applied a tenfold downsampling, the sampling period became $\Delta t = 0.01$ s. For the multiplicative random walk with linear anomaly dataset we applied a logarithmic difference as a preprocessing step to get rid of nonstationarity in the time series (Eq. 26).

$$y_t = \log(x_t) - \log(x_{t-1}) \tag{26}$$

where x is the original time series, log is the natural logarithm and y is the preprocessed time series.

In the case of TOF and LOF, time delay embedding was applied on the scalar time series. For the logistic map - tent map and - linear datasets the dynamics is well known and 1-dimensional, so $E = 3$ embedding dimension is enough to embed the signal. Also, $\tau = 1$ time-step was proper for an embedding delay. For the ECG dataset the dynamics naively seems to be approximately 2-dimensional, so we set $E = 3$, which may be enough to reconstruct the dynamics, also $\tau = 0.01$ s was set as embedding delay.

After embedding, the ROC AUC score was computed to find optimal neighborhood sizes in the $k \in \{1,..199\}$ range with the TOF and the LOF methods (Fig. 3 A).

As a next step of comparison, a screening over the anomaly-length parameter was performed and optimal F_1 score was registered for the TOF, LOF and brute force discord detection (Fig. 3 B)). More specifically, the F_1-score metrics, precision and recall were calculated on the simulated datasets in the function of event length parameter in the $[1,300]$ integer range for the discrete-time datsets and in the $[1,3000]$ integer range on the simulated ECG dataset. The embedding dimension was set to $E = 3$, and embedding delay $tau = 1$, the neighborhood size parameter was set to $k = 4$ in the case of TOF, and $k = 28$, 1, 99, 1 for LOF applied on the logistic map-tent map, logistic map-linear simulated ECG and random walk-linear datasets respectively. We applied the brute force discord discovery on the simulated datasets, and calculated the F_1 score in the function of neighborhood size and window length parameters respectively. The window length parameter were varied the same way we changed the event length parameter for TOF or the percentage of outliers for LOF.

We ran Senin's RRA algorithm on the simulated datasets for discord discovery with automated event length selection[8,9]. We set the maximal sliding window size to 200 time-steps for the discrete time simulations and to 2000 time-steps for the simulated ECG datasets. The $p_{aa} = 4$ was set according to the example script and the alphabet size was set to a=8.

To show that TOF finds unique events, we applied the algorithm on time series with multiple anomalies. We made no preprocessing on the dataset and the embedding parameters were set to $E = 3$ and $\tau = 1$. Also the neighborhood size was set to $k_{\text{TOF}} = 4$ and $k_{\text{LOF}} = 28$ for TOF and LOF respectively. We calculated the ROC AUC values for each simulated instance and plotted these values as the function of inter event interval (Fig. 4).

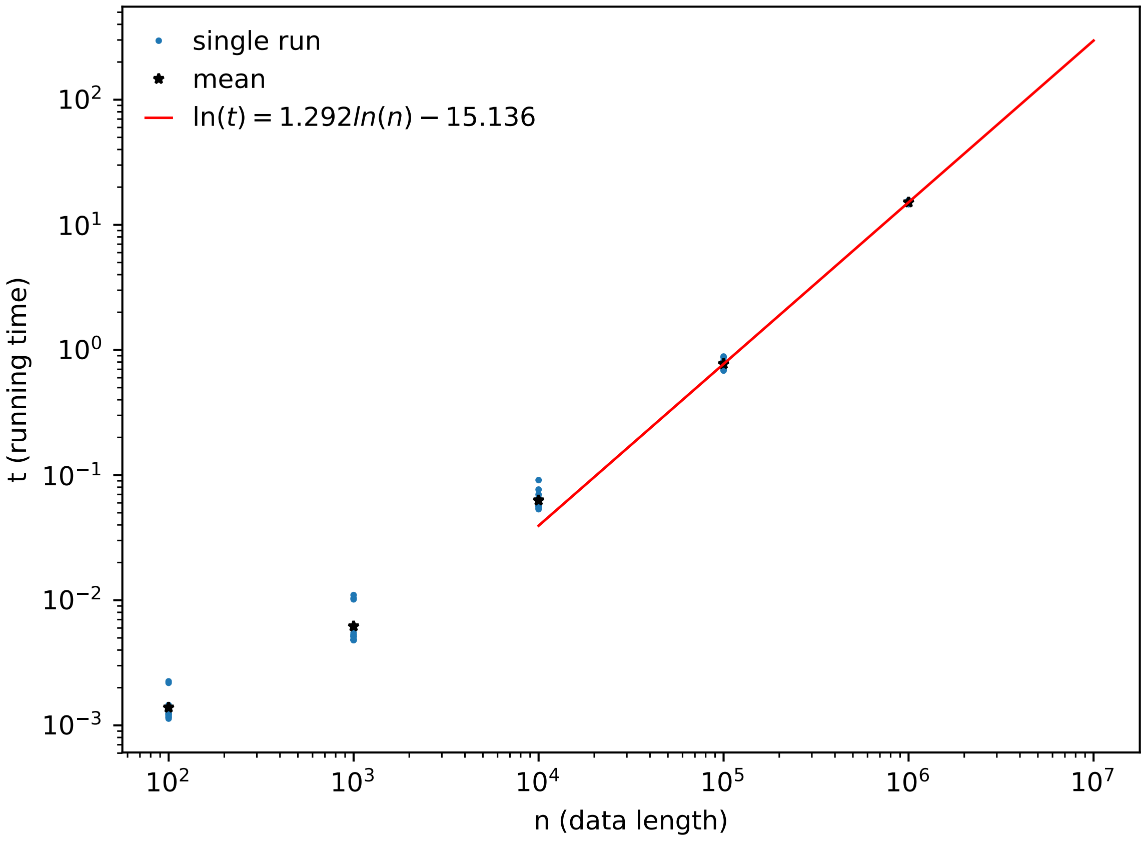

Figure S4. Running time as a function of time series length. Single runs (blue dots) and datalength-wise means (black stars) are shown along with the line fitted on the last two lengths (red line, ($d = 3$, $\tau = 1$, $k = 4$)

Computational complexity and running time

The current implementation of the TOF algorithm contains a time delay embedding, a kNN search, the computation of TOF score from the neighborhoods and threshold application. The time-limiting step is the neighbor-search, which uses the scipy cKDTree implementation of the kDTree algorithm[4]. The most demanding task is to build the tree data-structure; its complexity is $O(kn \log n)$[11] and the nearest neighbor search has $O(\log n)$ complexity.

We applied the TOF algorithm on random noise from $10^2 - 10^6$ sample size, 15 instances each ($d = 3$, $\tau = 1$, $k = 4$). The running-time on the longest tested dataset containing 10^6 points was $15,144 \pm 0.351$ secs (Fig. 4) on a laptop powered by Intel®Core™i5-8265U CPU.

We fit a line on the log-log plot where the data-lengths were $n = 10^5$ and $n = 10^6$. The following equation described the fitting line:

$$log(t) = 1.292 \ln(n) - 15.136 \tag{27}$$

.

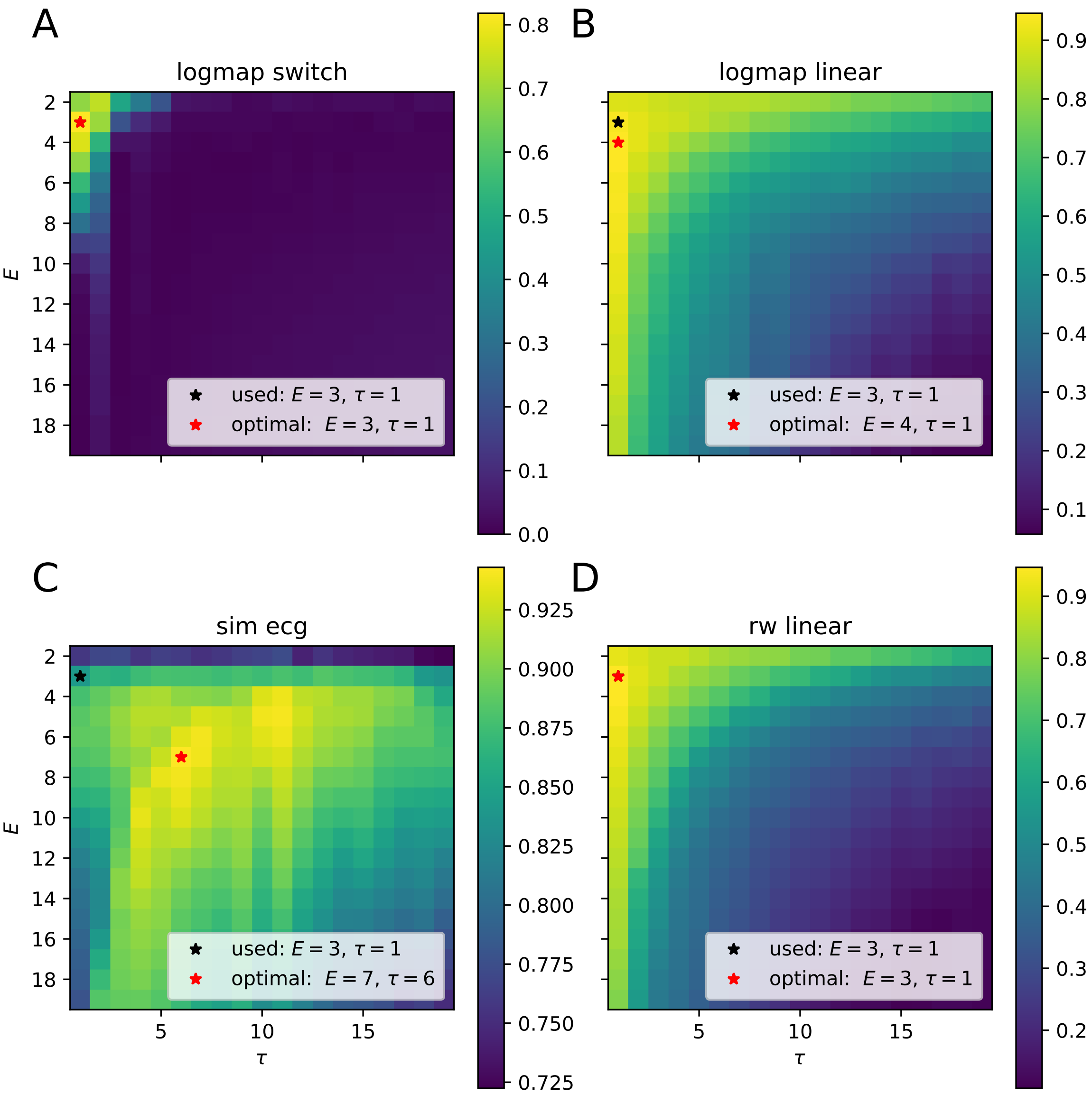

Figure S5. **F_1 score in the function of embedding dimension and embedding delay for the simulated datasets ($N = 15$).** **A** Logistic map with tentmap anomaly ($E^* = 3$, $\tau = 1$, $F_1^{max} = 0.818$), **B** logistic map with linear anomaly ($E^* = 4$, $\tau = 1$, $F_1^{max} = 0.946$), **C** simulated ECG with tachycardia ($E^* = 7$, $\tau = 6$, $F_1^{max} = 0.942$) and **D** random walk with linear anomaly ($E^* = 3$, $\tau = 1$, $F_1^{max} = 0.947$).

Embedding-parameter dependence

We investigated the parameter-dependence of TOF detection perfomance by measuring the F_1 score on a range of embedding dimension ($d \in \{2,..19\}$) and embedding delay ($\tau \in \{1,..19\}$) pairs, while keeping the threshold parameter fixed on the simulated datasets ($N = 15$ each, Fig. 5). The threshold parameter was set to 110 for the discrete-time datasets, and 1100 for the simulated ECG dataset.

We found that the performance was parameter-depedent, but near optimal parameters can be found in most cases with basic knowledge about the investigated system.

It is worth mentioning that the optimal and near-optimal parameter combinations traced out a hyperbola in the search space pointing a quazy-constant optimal embedding-window length specific to each dataset.

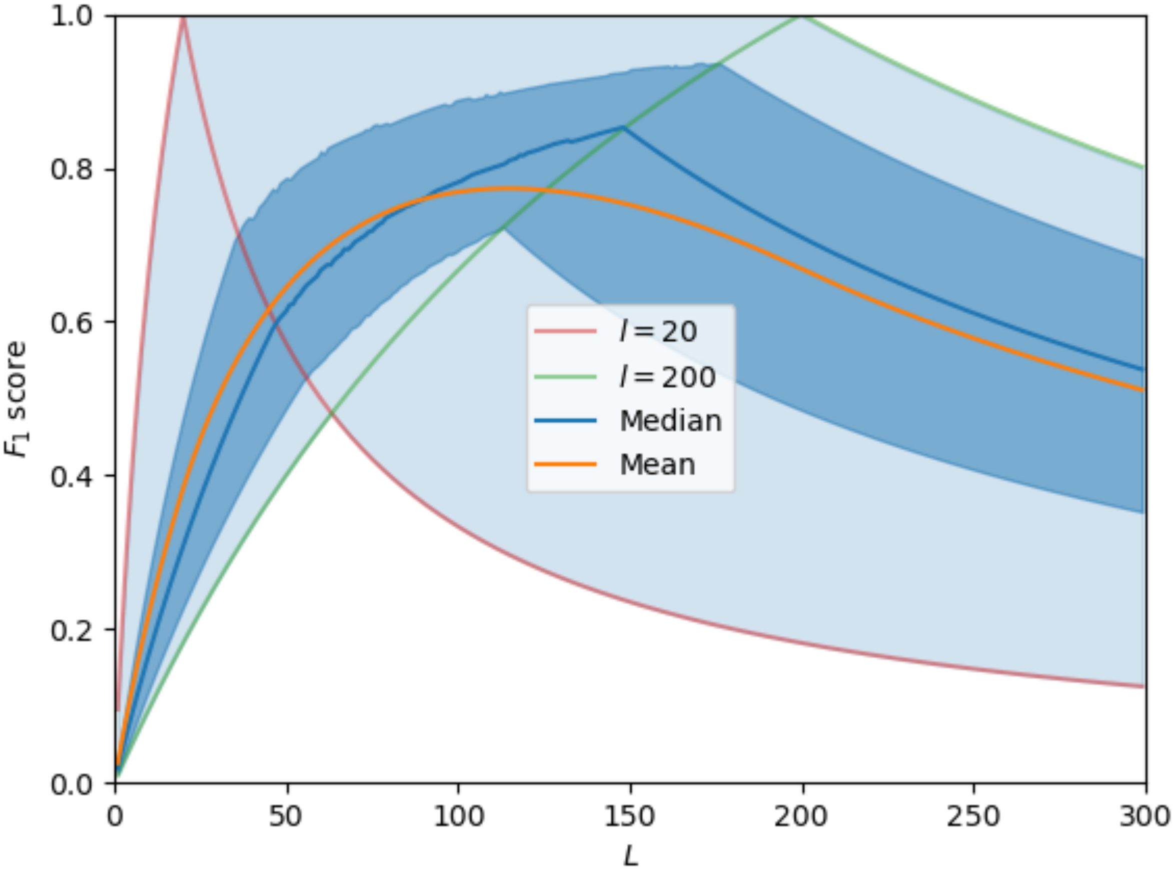

Figure S6. Simulated Expectation of the upper limit F_1 score for LOF and discord in the function of length parameter on the simulated data. The figure shows the F1 scores of simulated time series ($N = 10000$) with randomly varied anomaly length in the function of length parameter (L). The median (blue curve) and mean (yellow curve) of the F_1 scores is also marked on the figure. The shortest anomaly has the length of $l = 20$ (red curve) time-steps and the longest one is $l = 200$ time-steps long (green curve). These two curves mark the range of possible F_1 score values measured on the dataset (Blue shading) and to get some sense of the distribution, the inter-quartile range (strong shading) is also shown. The F_1 score strongly depends on the length parameter, the estimated maximum is at 114 timesteps, which is around the expected event length (110) of the simulated outlier segments.

Maximum expected F_1 score of the simulated dataset

When the event length is unknown, the maximal achievable F_1 score may be limited by the event length parameter.

We computed the maximal possible F_1 score given the length parameter of anomaly detection methods. We simulated N=10000 realizations of true event lengths drawn from a discrete uniform distribution over the [20, 200] range, and computed the maximum possible F_1 score metric given the length parameter (L) in the (1, 300) range. We took the L-wise mean and median of the sample and plotted the results (Fig. 6).

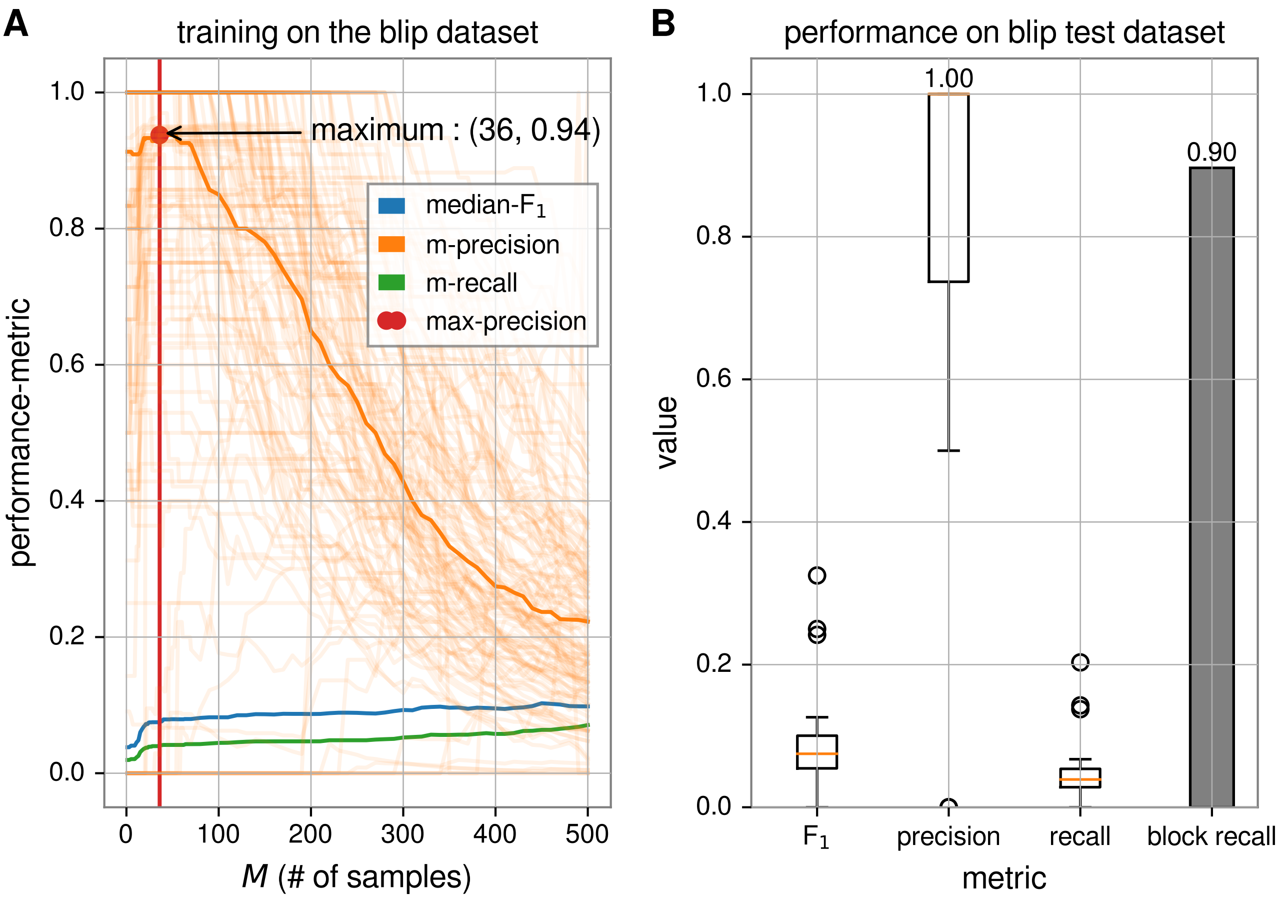

Figure S7. **TOF Results on the Gravity Spy blip dataset.** **A** The mean F_1 score, precision and recall metrics from the training set are shown ($N = 128$) and the maximal precision place ($M = 36$) selected to test evaluation. **B** The test ($N = 29$) F_1 score, precision,recall and block recall. The median precision is 1.00 and the block recall (hit rate) is 0.9.

Local Outlier Factor

The Local Outlier Factor[12] compares local density around a point (X) with the density around its neighbors (Eq. 28).

$$\mathrm{LOF}_k(X) = \frac{1}{|N_k(X)|} \sum_{o \in N_{k(X)}} \frac{\mathrm{lrd}_k(o)}{\mathrm{lrd}_k(X)} \tag{28}$$

Where $|N_k(X)|$ is the cardinality of the k-distance neighborhood of X, lrd_k is the local reaching density for k-neighborhood (see Breunig et al. [12] for details, Fig. S1).

Analysis of real-world data

Polysomnography dataset

We analysed a part of the first recording of the MIT-BIH polysomnographic database[13] on Physionet[14]. The ECG data was sampled at 250 Hz. A 160 s long segment was selected to be analysed, starting at 300 s of the recording. The embedding parameters were set by a manual procedure to $E_{\mathrm{TOF}} = 3$ and $\tau = 0.02$ s. The embedding delay was set according to the first zero-crossing of the autocorrelation function, embedding dimension was determined by an iterative embedding process, where the intrinsic dimensionality[15] of the dataset was measured for various embedding dimensions (Fig S9). The embedding dimension where the intrinsic dimensionality started to saturate was selected. For LOF, the embedding dimension was set higher ($E_{\mathrm{LOF}} = 7$), because the results became more informative about the apnea event. The neihgborhood size was set according to

simulation results; we used a smaller neighborhood for TOF ($k = 11$) and a large neighborhood for LOF ($k = 200$). Moreover we set the event length to $M = 5$ s for TOF, corresponding to 3.125% for LOF, which turned out to be a too loose condition. Therefore we used the more conservative 0.5% threshold for LOF to get more informative results.

Gravitational wave dataset

We analysed the 4096 Hz sampling rate strain data of the LIGO Hanford (H1) detector around the GW150914 merger event. The analysed 12 s recording starts 10 s before the event. We investigated the q transform spectrogram of the time series around the event at 5×10^{-4} s time resolution by using the gwpy python package[16]. Based on the spectrogram we applied 50-300 Hz bandpass filtering on the time series as a preprocessing step. Embedding parameters were selected manually (Fig. S10), by choosing the first minima of the autocorrelation function for the embedding delay ($\tau = 8$ sampling periods ≈ 1.95 ms) and then we selected the embedding dimension according to a manual procedure. Successive embedding of the time series into higher and higher dimensional space showed, that the intrinsic dimensionality of the dataset starts to deviate from the embedding dimension at $E = 6$. Thus, we set this latter value as embedding dimension for TOF. For LOF a higher embedding dimension ($E = 11$) led to informative results. We set the neighborhood sizes based on our experiences with the simulated datasets: smaller value was set for TOF ($k = 12$) and larger for LOF ($k = 100$). The event length was set to $M = 146$ ms for TOF as the visible length of the chirp on the spectrogram and 0.5 % for LOF. Also, a $w = 7$ widening window was applied on TOF detections.

Gravity Spy blip dataset

Data acquisiton: We downloaded randomly chosen blip events registered in the Gravity Spy[17] database from the GWOSC[18] servers using the gwpy python package[16]. Time series length was randomly chosen (0.15-2sec) around the blip events. The start time and duration of each event was acquired from the Gravity Spy metadata file, and a random-length pre and post segment were added to the event. After downloading the data, the data-files containing missing values were removed. At the end of the download and quality check steps, the training set contained $N = 128$ and the test set contained $N = 29$ blip time series.

Preprocessing and application of TOF: We bandpass-filtered the signals (100-300 Hz) with default parameters two times (mne.filter/filter_data function) and cropped the time series to get rid of distorted edges (200 timesteps). We applied time delay embedding ($d = 3$, $\tau = 1$) and applied TOF to predict anomalies in the function of the event-threshold in the 1-500 time-step range for the training data. We applied the TOF algorithm on the test set with optimal threshold parameter (see below).

Performance metrics: We calculated F_1 score, precision and recall values for the threshold range and we optimized the median precision value to select a threshold value ($M = 36$, precision= 0.94, Fig. S7). Furthermore, we computed the block recall metric on the test set, which measures the ratio of datasets in which TOF found points of blip events.

Training and test results: The metrics showed high median precision, low median recall and low median F_1 score for the training set (Fig. S7 A). On the test set, we applied TOF with the optimized threshold ($M = 36$) and got very high median precision (1.00, IQR:0.263) and high block recall (0.9) with low F_1 score and recall (Fig. S7 B).

LIBOR dataset

The monthly LIBOR dataset was analysed to identify interesting periods. As a preprocessing step, the first difference was applied for detrending purposes.

Optimal Embedding parameters were selected according to the minima of the relative entropy ($E = 3$, $\tau = 1$ month, Fig. S11-S12). The neighborhood size was set manually to $k_{TOF} = 5$ and $k_{TOF} = 30$ for TOF and LOF respectively. Also, the event length was $M = 30$ for TOF and the threshold was set to 18.86 % for LOF. Also, a widening window $w = 3$ was applied on TOF detections.

Additional Tables and Figures

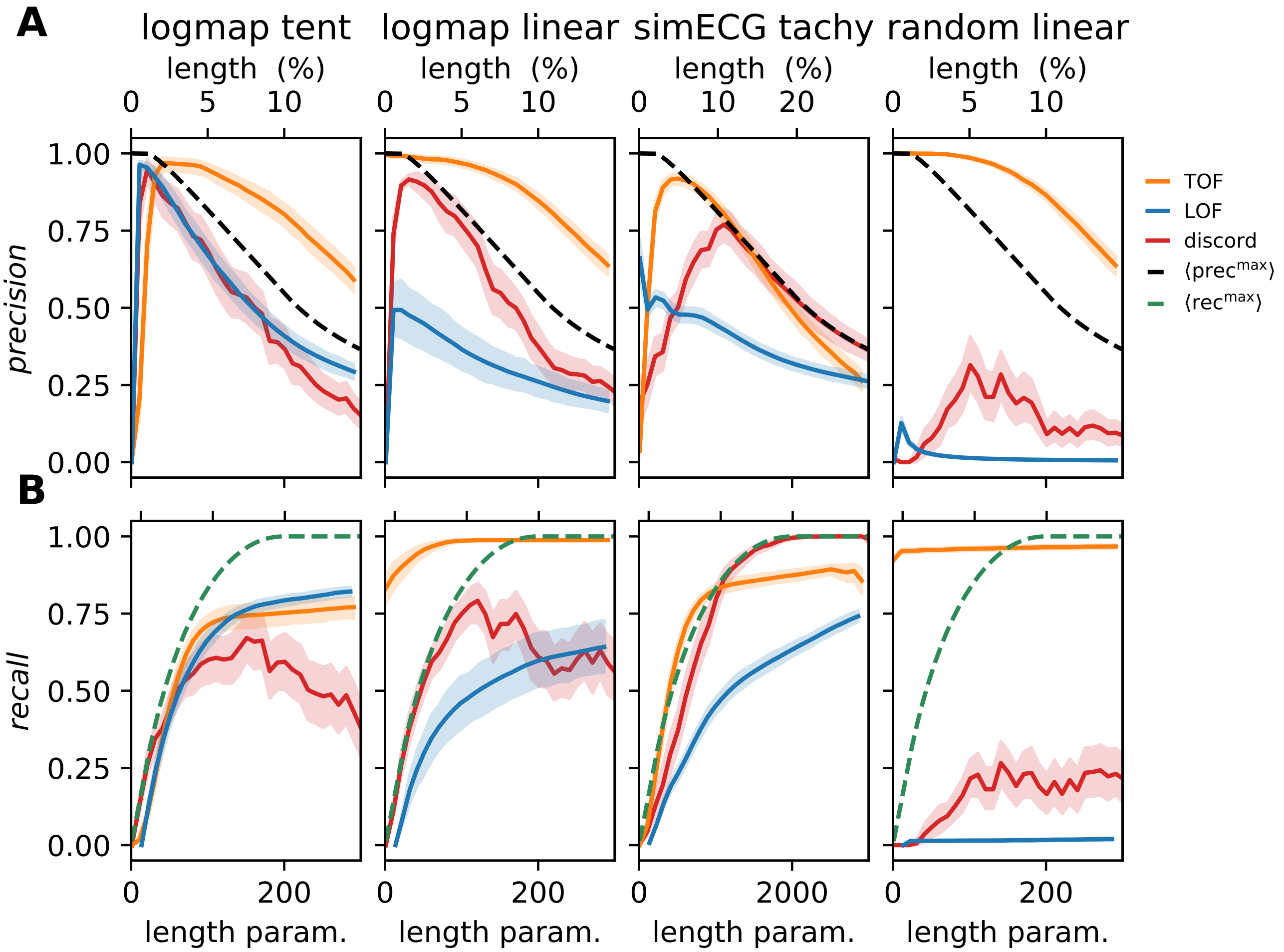

Figure S8. Precision and recall and its dependence on the expected length parameters of the three methods on four different test datasets. Upper row mean precision, lower row mean recall and SD, over n=100 test dataseries for all four dataset types. Orange line: TOF, Blue line: LOF, Red line: discord, black-dashed: mean maximal precision for LOF and discord methods, green-dashed: mean maximal recall for LOF and discord. **A** Precision score reached by the methods on the test data-sets. **B** Recall score reached by the methods on the test data-sets.

Table S1. State space densities and LOF values within normal and anomalous activity. Median and median absolute difference of the density of the points and LOF values in the reconstructed state space are shown, calculated from the distance of the 20 nearest neighbors. The density of the anomaly was significantly lower than the density generated by normal activity in two cases: the tent map anomaly in logistic backgroud the tachycardia within the normal heart rhythm. These cases also resulted in higher LOF values of anomalies. While the density of the linear anomaly segments was not significantly different from the logistic background, the linear anomalies generated much higher density than the normal random walk time series after detrending. Correspondingly, LOF values were not significantly higher in these two cases within the anomaly than in the normal activity.

dataset	Density		LOF	
	Normal	Anomaly	*Normal*	Anomaly
logmap tent	95.759 ± 12.070	11.606 ± 1.146	1.039 ± 0.010	3.424 ± 1.990
logmap linear	95.190 ± 9.305	97.413 ± 51.289	1.040 ± 0.012	1.398 ± 0.451
sim ECG tachy	10146 ± 2227	168.370 ± 38.699	1.106 ± 0.022	1.264 ± 0.227
randwalk linear	197.919 ± 3.866	52590 ± 61527	1.623 ± 0.661	1.872 ± 0.920

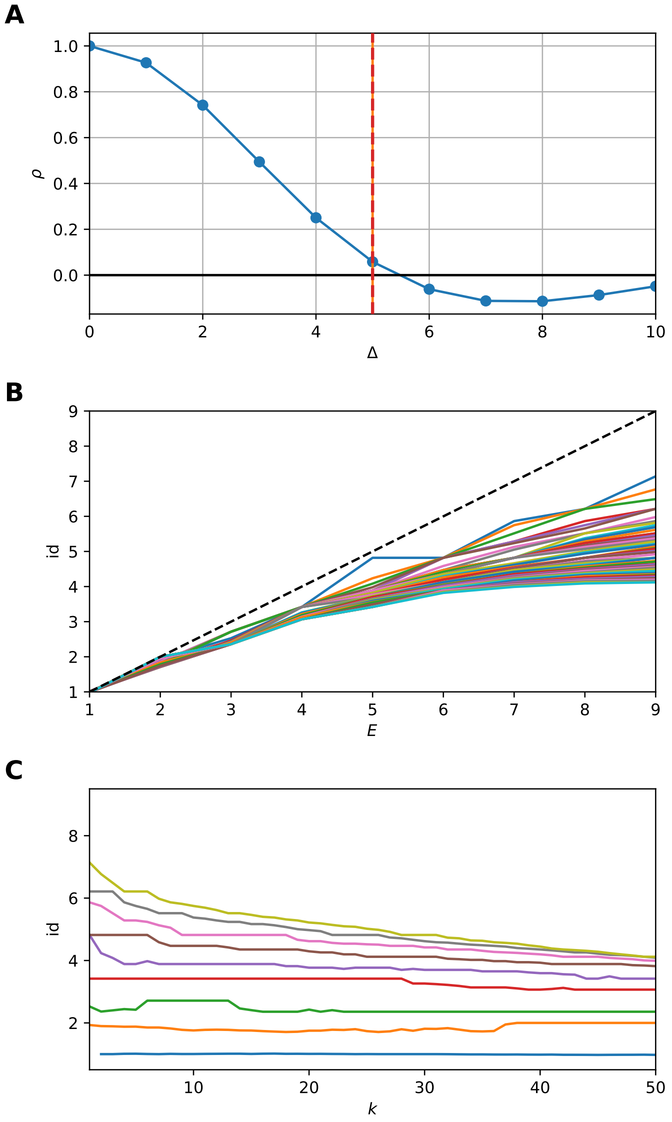

Figure S9. **Embedding parameter selection for the polysomnography data. A** Embedding delay was selected ($\tau = 5$ sampling period) according to the first zerocrossing of the autocorrelation function. The timeshift ensures the most linearly independent axes in reconstructed state space. **B** The intrinsic dimensionality is measured as a function of embedding dimension (E) for various neighborhood sizes. The dimension-estimates start to deviate from the diagonal at $E = 3$. **C** Intrinsic dimensionality in the function of neighborhood size (k) for various embedding dimensions.

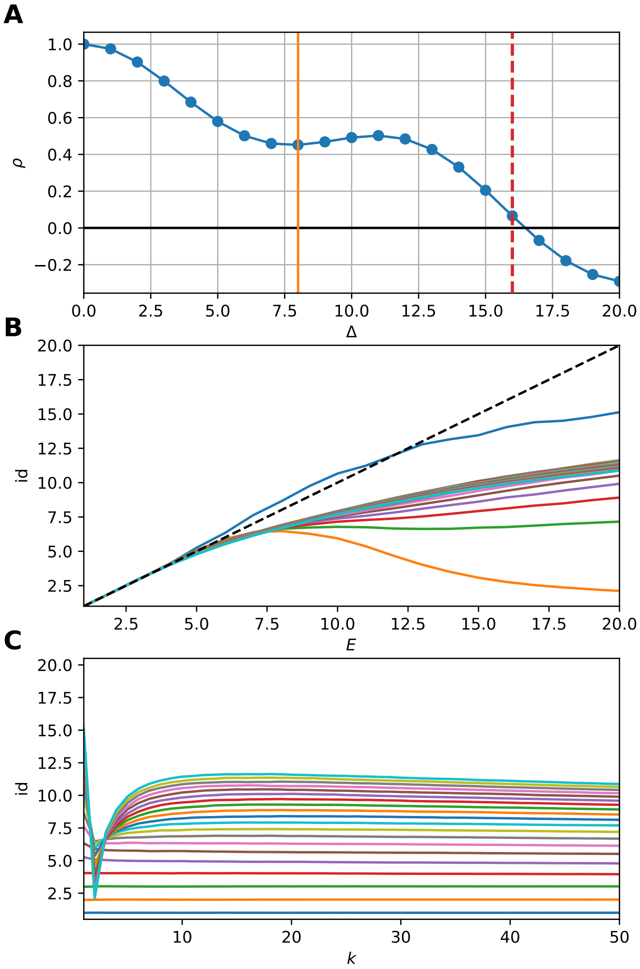

Figure S10. **Embedding parameter selection for the gravitational wave data. A** Embedding delay was selected ($\tau = 8$ sampling period) according to the first minima of the autocorrelation function. The first zeropoint was between 16 and 17 sampling periods. **B** The intrinsic dimensionality is measured as a function of embedding dimension (E) for various neighborhood sizes. The dimension estimates start to deviate from the diagonal at $E = 5$. **C** Intrinsic dimensionality as a function of neighborhood size (k) for various embedding dimensions.

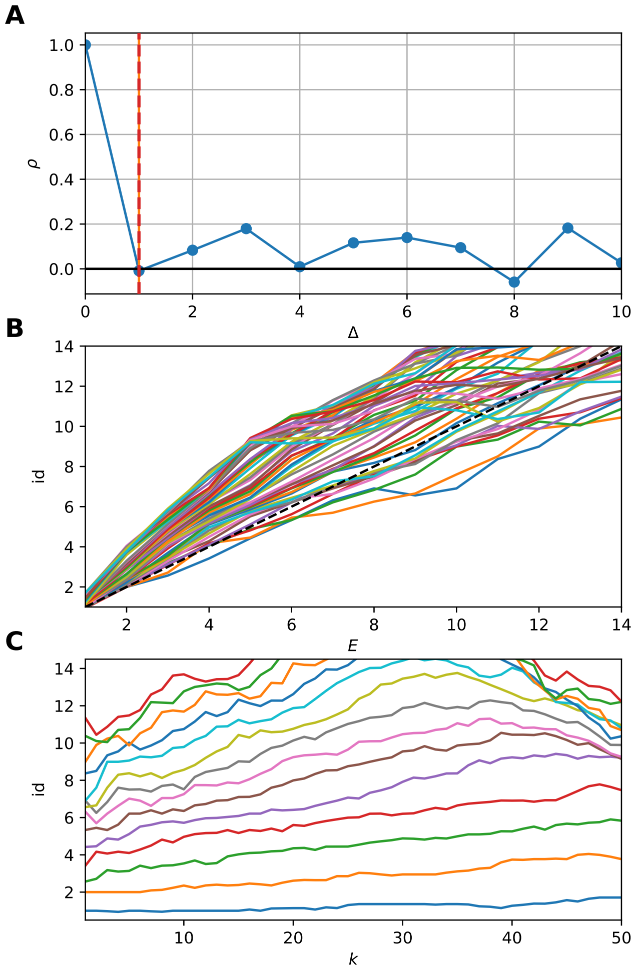

Figure S11. **Autocorrelation and intrinsic dimension measurement of the preprocessed LIBOR time series.**

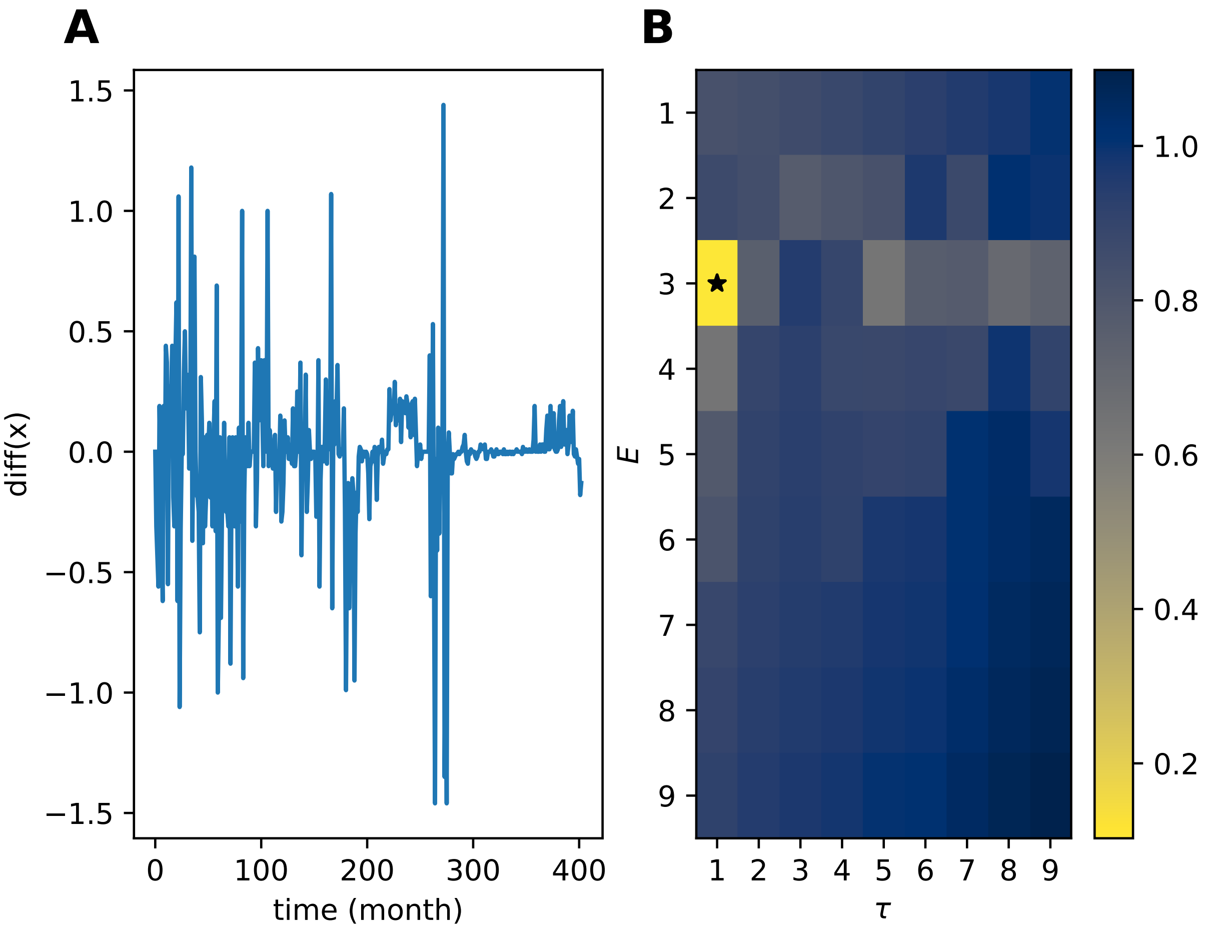

Figure S12. **Preprocessing and embedding parameter selection for the LIBOR time series with differential entropy.** (left) The discrete time derivative of the original time series was calculated to detrend the data (right). The minima of the entropy landscape marks the optimal embedding parameters ($E = 3, \tau = 1$ timestep).

References

1. Rhodes, C. & Morari, M. The false nearest neighbors algorithm: An overview. *Comput. & Chem. Eng.* DOI: 10.1016/S0098-1354(97)87657-0 (1997).
2. Krakovská, A., Mezeiová, K. & Budáčová, H. Use of False Nearest Neighbours for Selecting Variables and Embedding Parameters for State Space Reconstruction. *J. Complex Syst.* **2015**, 1–12, DOI: 10.1155/2015/932750 (2015).
3. Gautama, T., Mandic, D. P. & Van Hulle, M. M. A differential entropy based method for determining the optimal embedding parameters of a signal. In *ICASSP, IEEE International Conference on Acoustics, Speech and Signal Processing - Proceedings*, vol. 6, 29–32, DOI: 10.1109/icassp.2003.1201610 (2003).
4. Bentley, J. L. Multidimensional binary search trees used for associative searching. *Commun. ACM* **18**, 509–517, DOI: 10.1145/361002.361007 (1975).
5. Virtanen, P. *et al.* SciPy 1.0: Fundamental Algorithms for Scientific Computing in Python. *Nat. Methods* **17**, 261–272, DOI: https://doi.org/10.1038/s41592-019-0686-2 (2020).
6. Pedregosa, F. *et al.* Scikit-learn: Machine learning in Python. *J. Mach. Learn. Res.* **12**, 2825–2830 (2011).
7. Keogh, E., Lin, J. & Fu, A. HOT SAX: Efficiently finding the most unusual time series subsequence. In *Proceedings - IEEE International Conference on Data Mining, ICDM*, DOI: 10.1109/ICDM.2005.79 (2005).
8. Senin, P. *et al.* Time series anomaly discovery with grammar-based compression. *EDBT 2015 - 18th Int. Conf. on Extending Database Technol. Proc.* 481–492, DOI: 10.5441/002/edbt.2015.42 (2015).
9. Senin, P. jmotif. *https://github.com/jMotif/jmotif-R* (2020).
10. Ryzhii, E. & Ryzhii, M. A heterogeneous coupled oscillator model for simulation of ECG signals. *Comput. Methods Programs Biomed.* **117**, 40–49 (2014).
11. Brown, R. A. Building a Balanced k-d Tree in $O(kn\log n)$ Time. *J. Comput. Graph. Tech. (JCGT)* **4**, 50–68 (2015).
12. Breunig, M. M., Kriegel, H.-P., Ng, R. T. & Sander, J. LOF: Identifying density-based local outliers. *SIGMOD Rec. (ACM Special Interest Group on Manag. Data)* DOI: 10.1145/335191.335388 (2000).
13. Ichimaru, Y. & Moody, G. B. Development of the polysomnographic database on CD-ROM. *Psychiatry Clin. Neurosci.* DOI: 10.1046/j.1440-1819.1999.00527.x (1999).
14. Goldberger, A. L. *et al.* PhysioBank, PhysioToolkit, and PhysioNet: components of a new research resource for complex physiologic signals. *Circulation* (2000).
15. Massoud Farahmand, A., Szepesvári, C. & Audibert, J.-Y. Manifold-adaptive dimension estimation. In *Proceedings of the 24th international conference on Machine learning - ICML '07*, 265–272 (ACM Press, New York, New York, USA, 2007).
16. Macleod, D. *et al.* gwpy/gwpy: 1.0.1, DOI: 10.5281/zenodo.3598469 (2020).
17. Zevin, M. *et al.* Gravity spy: integrating advanced ligo detector characterization, machine learning, and citizen science. *Class. Quantum Gravity* **34**, 064003, DOI: 10.1088/1361-6382/aa5cea (2017).
18. Abbott, R. *et al.* Open data from the first and second observing runs of Advanced LIGO and Advanced Virgo (2019). 1912.11716.
